# Pyruvate kinase M2 sustains cardiac mitochondrial quality surveillance in septic cardiomyopathy by regulating prohibitin 2 abundance via S91 phosphorylation

**DOI:** 10.1007/s00018-024-05253-9

**Published:** 2024-06-10

**Authors:** Yingzhen Du, Jialei Li, Zhe Dai, Yuxin Chen, Yao Zhao, Xiaoman Liu, Tian Xia, Pingjun Zhu, Yijin Wang

**Affiliations:** 1 The Second Medical Center & National Clinical Research Center for Geriatric Diseases, Chinese PLA General Hospital, Medical School of Chinese PLA, Beijing, China; 2 School of Medicine, Southern Medical University, Guangzhou, Guangdong China; 3 Department of Clinical Laboratory Medicine, The First Medical Centre, Medical School of Chinese People’s Liberation Army, Beijing, China; 4 Xianning Medical College, Hubei University of Science & Technology, Xianning, China

**Keywords:** PKM2, PHB2, MQC, Septic cardiomyopathy, Mitochondria

## Abstract

The endogenous mitochondrial quality control (MQC) system serves to protect mitochondria against cellular stressors. Although mitochondrial dysfunction contributes to cardiac damage during many pathological conditions, the regulatory signals influencing MQC disruption during septic cardiomyopathy (SC) remain unclear. This study aimed to investigate the involvement of pyruvate kinase M2 (PKM2) and prohibitin 2 (PHB2) interaction followed by MQC impairment in the pathogenesis of SC. We utilized LPS-induced SC models in PKM2 transgenic (PKM2^*TG*^) mice, PHB2^*S91D*^-knockin mice, and PKM2-overexpressing HL-1 cardiomyocytes. After LPS-induced SC, cardiac PKM2 expression was significantly downregulated in wild-type mice, whereas PKM2 overexpression in vivo sustained heart function, suppressed myocardial inflammation, and attenuated cardiomyocyte death. PKM2 overexpression relieved sepsis-related mitochondrial damage via MQC normalization, evidenced by balanced mitochondrial fission/fusion, activated mitophagy, restored mitochondrial biogenesis, and inhibited mitochondrial unfolded protein response. Docking simulations, co-IP, and domain deletion mutant protein transfection experiments showed that PKM2 phosphorylates PHB2 at Ser91, preventing LPS-mediated PHB2 degradation. Additionally, the A domain of PKM2 and the PHB domain of PHB2 are required for PKM2-PHB2 binding and PHB2 phosphorylation. After LPS exposure, expression of a phosphorylation-defective PHB2^S91A^ mutant negated the protective effects of PKM2 overexpression. Moreover, knockin mice expressing a phosphorylation-mimetic PHB2^S91D^ mutant showed improved heart function, reduced inflammation, and preserved mitochondrial function following sepsis induction. Abundant PKM2 expression is a prerequisite to sustain PKM2-PHB2 interaction which is a key element for preservation of PHB2 phosphorylation and MQC, presenting novel interventive targets for the treatment of septic cardiomyopathy.

**Supplementary Information:**

The online version contains supplementary material available at 10.1007/s00018-024-05253-9.

## Introduction

As a severe complication of sepsis, septic cardiomyopathy (SC) manifests as reversible myocardial depression in patients with septic shock. Clinical features of SC include left ventricular dilation, decreased myocardial contractile function, and increased end-diastolic ventricular volume. Inflammatory injury, aberrant immune response, and cytokine storm have been proposed as the molecular bases of decreased myocardial function during sepsis-induced cardiomyopathy [1, 2]. However, findings from a randomized study in patients with virus-negative inflammatory cardiomyopathy suggest that immunosuppressive strategies may not provide additional clinical benefits for patients with SC [3, 4]. A growing body of evidence highlights the functional significance of mitochondrial damage in the pathogenesis of SC. Increased mitochondrial fission compromises cardiomyocyte energy status, leading to impaired myocardial contraction [5]. Additionally, defective mitophagy contributes to oxidative stress and thus mitochondria-dependent apoptosis in the myocardium [6, 7]. Decreased mitochondrial metabolism, reflected by impaired mitochondrial respiration, has been found to be closely associated with myocardial swelling and inflammation [8]. Such evidence suggests multiple mechanisms linking mitochondrial dysfunction to the pathogenesis of SC. However, the upstream mediators and signal transduction mechanisms responsible for inducing mitochondrial abnormalities in cardiomyocytes during sepsis remain incompletely characterized.

Mitochondrial quality control (MQC) is an inherent protective mechanism that regulates mitochondrial dynamics, mitophagy, biogenesis, and the mitochondrial unfolded protein response (mtUPR) to maintain normal mitochondrial morphology and function [9–13]. Our previous studies have reported the beneficial effects of MQC in the setting of cardiac ischemia-reperfusion injury (IRI) [14, 15]. In IRI-challenged cardiac cells with preserved MQC, mitochondrial potential was maintained, mitochondrial oxidative stress was neutralized, mitochondrial permeability transition pore (mPTP) opening was inhibited, and mitochondria-induced apoptosis was suppressed [14]. Such a scenario is supported by a recent study in a mouse model of type-2 diabetes [16], which delineated the cardioprotective impact of MQC on diabetic cardiomyopathy via upregulating mitochondrial respiration, accelerating mitochondrial ATP production, and reducing mitochondrial ROS leakage. By comparison, defective MQC is regarded as a contributory factor augmenting vanadium-mediated cardiovascular toxicity, possibly through inducing oxidative stress, mitochondrial vacuolar degeneration, and cardiomyocyte apoptosis [2, 17]. Despite this evidence, the specific alterations in MQC occurring during SC, as well as their underlying mechanisms, have not been entirely clarified.

Prohibitin 2 (PHB2) assembles at the inner mitochondrial membrane to form a supra-macromolecular structure that serves to maintain mitochondrial integrity, fusion/fission balance [18], biogenesis [19], and mitophagy [20, 21]. Evidence strongly indicates that PHB2 plays a pivotal role in coordinating the mitochondrial response to various stressors by regulating crucial aspects such as mitochondrial redox biology, mtDNA stability, membrane integrity, and respiratory activity [20–23]. While these results suggest that PHB2 functions as an essential MQC mediator, the impact of potential changes in PHB2 expression or stability in SC has not been determined.

Pyruvate kinase M2 (PKM2), one of the rate-limiting enzymes of glycolysis, critically affects various aspects of mitochondrial bioenergetics. PKM2 assembles in dimeric and tetrameric conformations. Tetrameric PKM2 has pyruvate kinase (PK) activity and catalyzes the production of pyruvate by phosphoenolpyruvate (PEP) [24]. Dimeric PKM2 has lower PK activity and exerts also non-glycolytic enzymatic functions, acting for instance as a transcription factor and a protein kinase [24]. Importantly, PKM2 may show ample intracellular distribution, e.g. attached to mitochondria, to the endoplasmic reticulum, and translocated to the nucleus [25]. Although mitochondrial dynamics [26], mitophagy [27], and biogenesis [28] have been reported to be controlled by PKM2, such influence has not been evaluated in the setting of SC. Considering the beneficial effects offered by PKM2 overexpression on heart failure [29] and myocardial infarction [30], we asked whether SC may be alleviated by PKM2 through stabilizing PHB2 and supporting MQC in cardiomyocytes.

## Results

### Downregulated PKM2 expression contributes to myocardial injury in SC

**Fig. 1:**
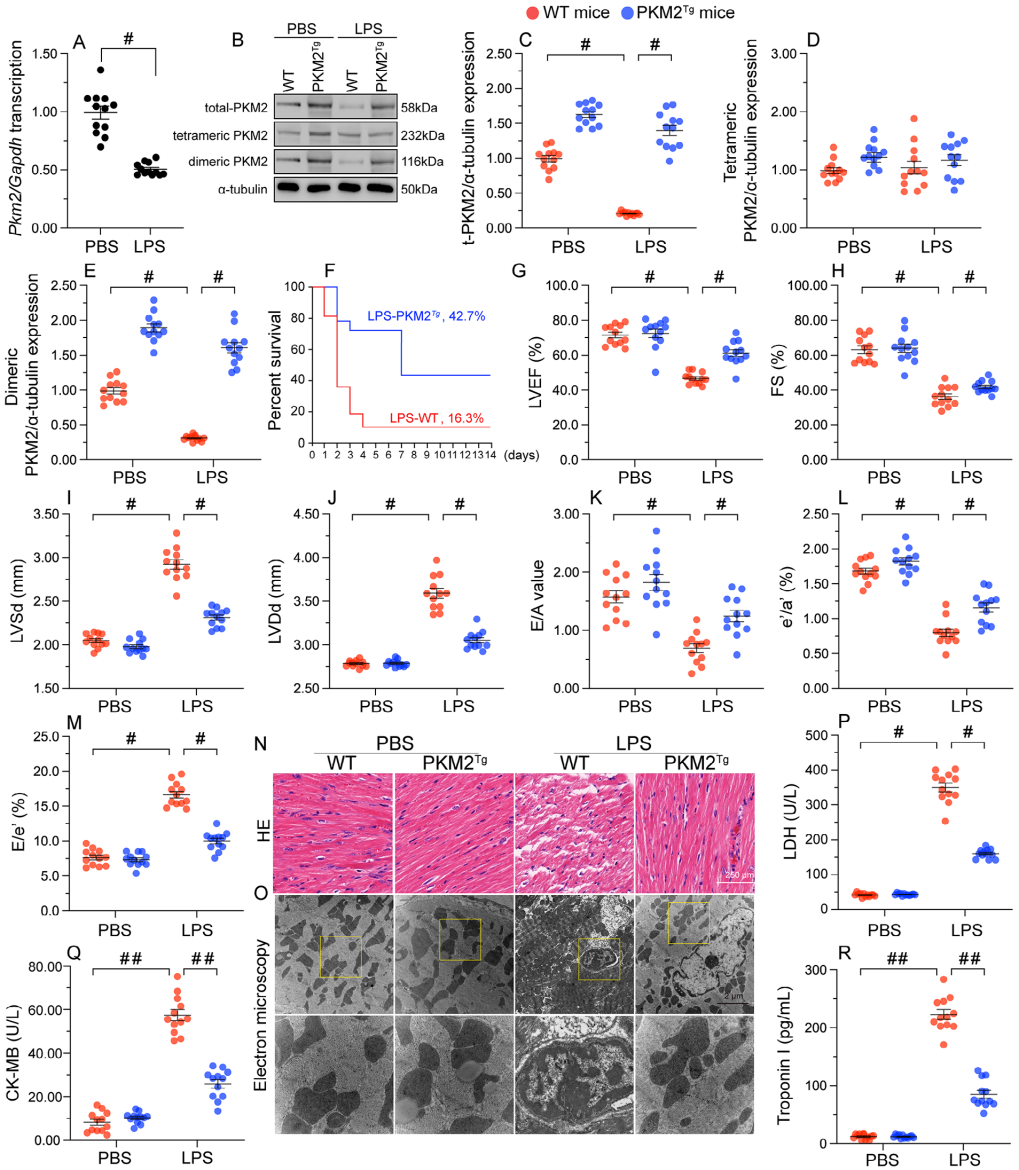
PKM2 downregulation contributes to myocardial injury in SC. PKM2 transgenic (PKM2^*Tg*^) mice and wild-type (WT) mice aged 8–10 weeks were injected intraperitoneally with 10 mg/kg LPS to induce SC. In vivo measurements were performed after 24 h later, and mice administered an equal volume of phosphate buffer saline served as controls. **(A)** Analysis of transcriptional levels of cardiac *Pkm2* in WT mice by qPCR. **(B-E)** Western blot analysis of total PKM2, dimeric PKM2, and tetrameric PKM2 protein expression in mouse heart tissues. **(F)** Survival analysis of control and PKM-overexpressing mice after SC induction. **(G-M)** Assessment of cardiac function in WT or PKM2^*Tg*^ mice by echocardiography. LVDd, left ventricular diastolic dimension; LVDs, left ventricular systolic dimension; IVS, interventricular septum thickness; E/A, ratio of early to late transmitral flow velocities; FS, ratio of left ventricular fractional shortening. **(N)** HE staining of myocardial tissue. **(O)** Representative images of electron microscopy of heart tissues. Yellow arrows indicate disorganized muscle fibers and deformed mitochondria with edematous cristae. **(P-R)** ELISA-based analysis of LDH, TnI, and CK-MB levels in mice sera. Values are presented as mean ± SEM. For in vivo data, *n* = 6 mice per group. For in vitro data, *n* = 4 independent experiments. #*p* < 0.05, and ##*p* < 0.01

To assess potential alterations in PKM2 expression during SC, qPCR and western blots were performed using heart tissues from LPS-treated mice. Both PKM2 mRNA transcription (Fig. 1A) and protein expression (Fig. 1B and C) were significantly downregulated compared to control tissues from WT mice. Importantly, upon LPS treatment, the levels of tetrameric PKM2 in cardiac tissue of WT mice remained unchanged, whereas a significant reduction was observed in the levels of dimeric PKM2 (Fig. 1D and E). This suggests that LPS mainly suppresses the content of dimeric PKM2 in the heart. To evaluate the potential cause-effect of PKM2 downregulation on myocardial injury during SC, we generated PKM2 transgenic (PKM2^*Tg*^) mice [31]. PKM2^*Tg*^ mice were born and developed normally with no detectable pathological symptoms, and showed ordinary phenotypes, lifespan, body weight, and heart function until 24 months of age. Western blots showed that cardiac levels of total PKM2 were similar, while those of tetrameric and dimeric PKM2 were slightly increased, in PKM2^*Tg*^ compared to WT mice (Fig. 1D and E).

Compared to WT mice, the mortality of PKM2^*Tg*^ mice was significantly reduced after SC induction (Fig. 1F). Moreover, upon LPS challenge, cardiac function assessed by echocardiogram, was impaired in WT mice and largely preserved in PKM2^*Tg*^ mice (Fig. 1G and M). Specifically, compared with WT mice, PKM2 overexpression markedly improved cardiac systolic function, as evidenced by normalized LVEF, FS, and LVSd after LPS administration (Fig. 1G and I). Similarly, alterations related to ventricular dilation were also ameliorated in PKM2^*Tg*^ mice compared with WT mice (Fig. 1J and M). To assess the presence of cardiac functional abnormalities associated with myocardial structural disorder, HE staining and electron microscopy (EM) were employed to detect myocardial morphology and cardiomyocyte ultrastructure. As expected, histological analysis indicated myocardial swelling in WT heart tissues accompanied by disorganized muscle fibers, vacuolated cardiomyocytes, and deformed mitochondria with edematous cristae (Fig. 1N and O). However, these structural derangements were not observed in LPS-challenged PKM2^*Tg*^ mice (Fig. 1N and O).

Myocardial injury is also featured by increased levels of myocardial damage biomarkers such as TnI, CK-MB, and LDH. After exposure to LPS, the concentrations of TnI, CK-MB, and LDH in sera were rapidly upregulated in WT mice and drastically relieved in PKM2^*Tg*^ mice (Fig. 1P and R). These results highlight a cardioprotective role of PKM2 in the setting of SC.

### PKM2 overexpression reduces myocardial inflammation in SC

**Fig. 2:**
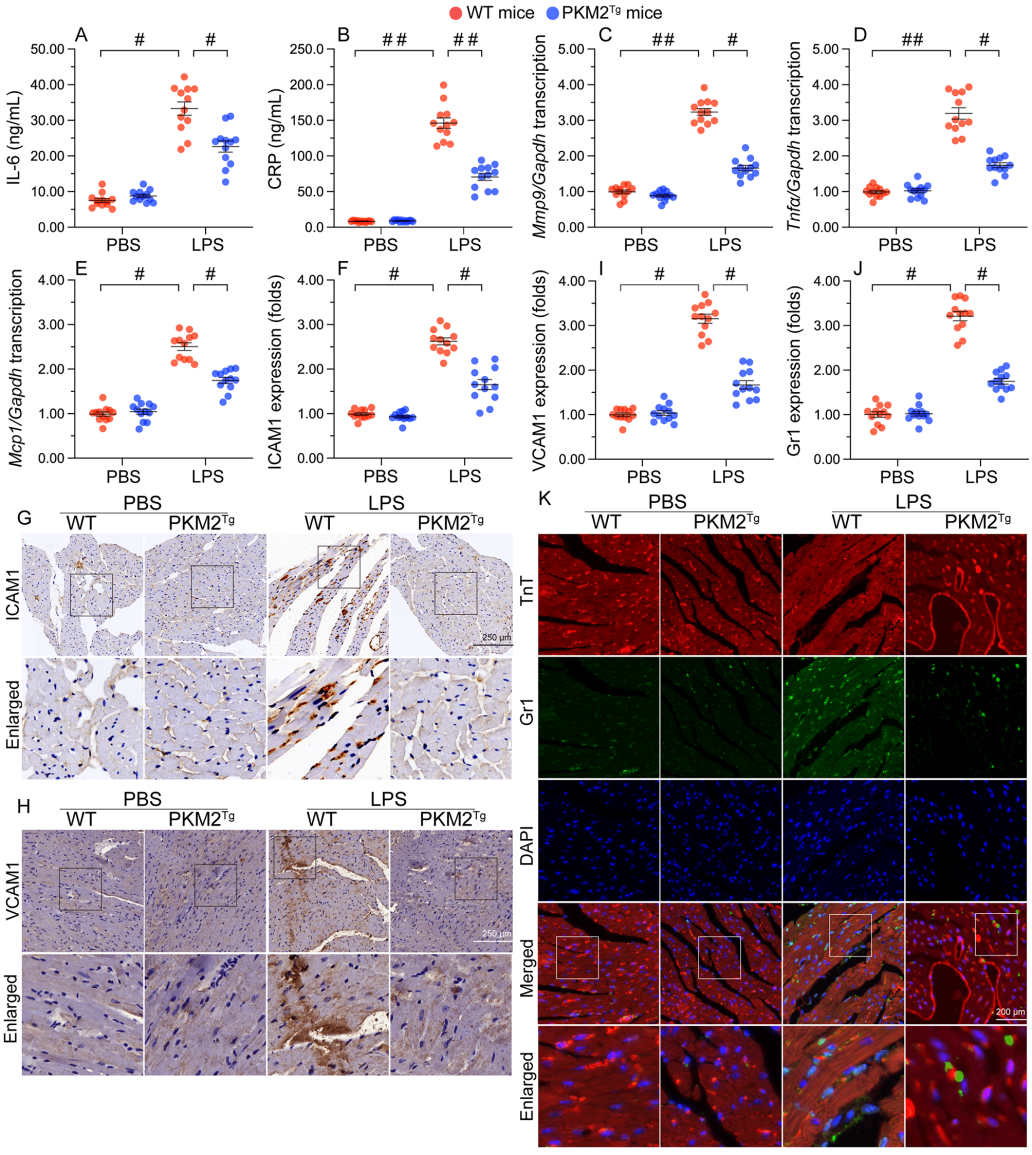
PKM2 overexpression reduces LPS-mediated myocardial inflammation. PKM2 transgenic (PKM2^*Tg*^) mice and wild-type (WT) mice aged 8–10 weeks were injected intraperitoneally with 10 mg/kg LPS to induce SC. In vivo measurements were performed after 24 h later, and mice administered an equal volume of phosphate buffer saline served as controls. **(A, B)** ELISA-based measurement of IL-6 and CRP levels in mouse serum. **(C-E)** Transcriptional analysis of cardiac *Mmp9*, *Mcp1*, and *Tnfα* expression in WT or PKM2^*Tg*^ mice by qPCR. **(F, G)** Quantification and representative images of immunohistochemical staining of ICAM1 in heart tissues. **(H, I)** Quantification and representative images of immunohistochemical staining of VCAM1 in heart tissues. **(J, K)** Immunofluorescent staining of Gr-1-positive neutrophils in heart tissues. Values are presented as mean ± SEM. For in vivo data, *n* = 6 mice per group. For in vitro data, *n* = 4 independent experiments. #*p* < 0.05, and ##*p* < 0.01

Abnormal inflammation has been identified as a core pathological factor aggravating myocardial damage under septic conditions. Thus, we sought to investigate whether PKM2 overexpression is capable to attenuate myocardial inflammatory reactions during sepsis. ELISA assay revealed a significant upregulation of IL-6 and CRP concentrations in the serum of WT mice following LPS treatment, whereas LPS-treated PKM2^*Tg*^ mice exhibited reduced levels of IL-6 and CRP (Fig. 2A and B). Besides, the transcriptions of pro-inflammation factors, such as *Mmp9*, *Mcp1* and *Tnfα*, were obviously elevated in WT heart tissue but maintained at near normal levels in PKM2-overexpressed mice after LPS treatment (Fig. 2C and E). Parallel increased pro-inflammatory cytokines and factors, adhesion proteins, i.e. ICAM1 (Fig. 2F and G) and VCAM1 (Fig. 2H and I), was also observed in heart tissues in WT mice but only remarkably recovered in PKM2^*Tg*^ mice. Accordingly, immunofluorescence showed that Gr-1-positive neutrophils were recruited into the myocardium of LPS-treated WT, but not PKM2^*Tg*^, mice (Fig. 2J and K). These findings indicate that PKM2 overexpression elicits an anti-inflammatory action in SC.

### PKM2 overexpression preserves cardiomyocyte viability during SC

**Fig. 3:**
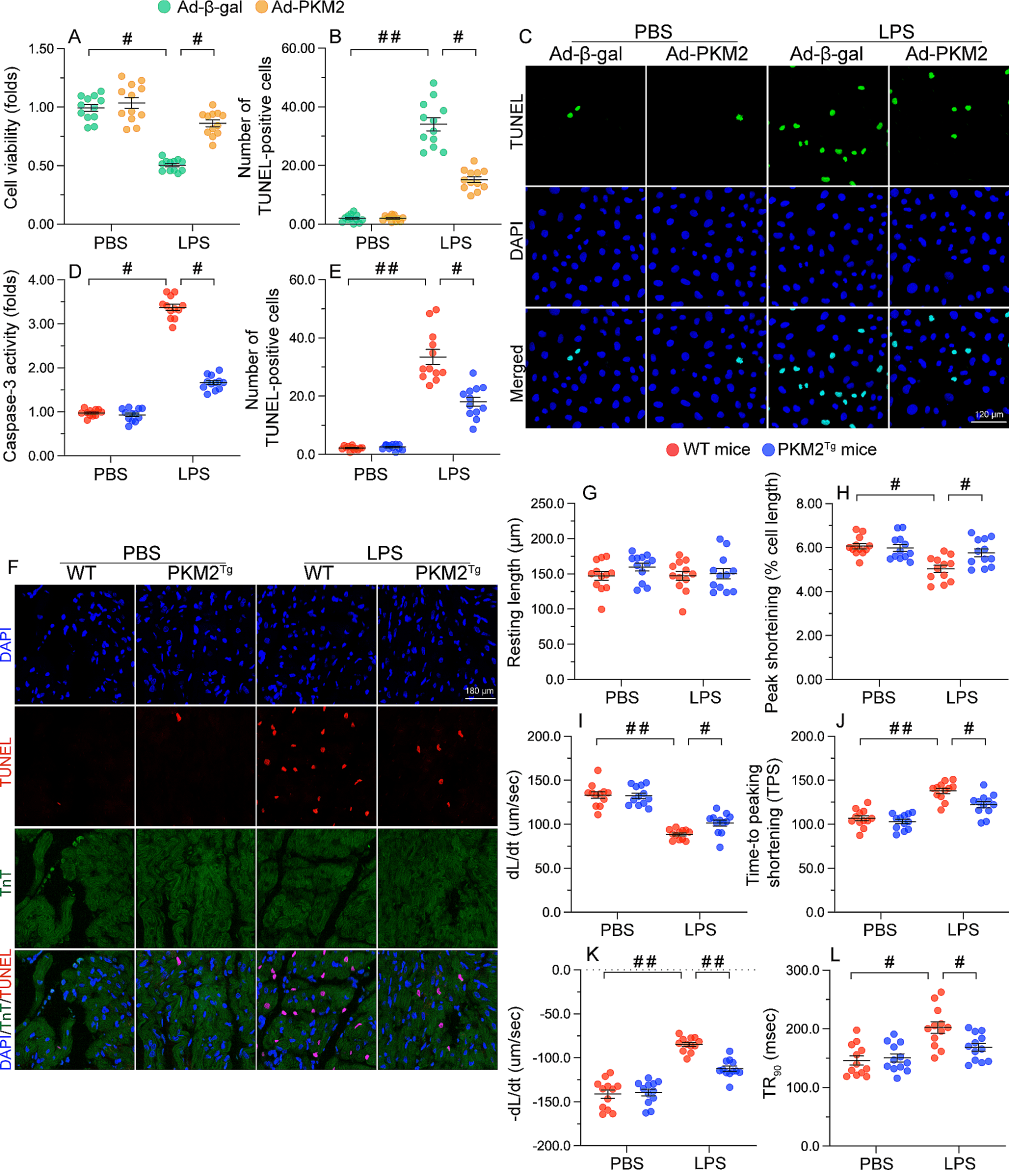
PKM2 overexpression preserves cardiomyocyte viability after septic insult. PKM2 transgenic (PKM2^*Tg*^) mice and wild-type (WT) mice aged 8–10 weeks were injected intraperitoneally with 10 mg/kg LPS to induce SC. In vivo and *ex-vivo* measurements were performed after 24 h later, and mice administered an equal volume of phosphate buffer saline served as controls. Immortalized mouse cardiac muscle HL-1 cells were treated with 10 µg/mL of LPS for 24 h to simulate SC in vitro. Cells treated with an equal volume of phosphate buffer saline were used as controls. Before LPS treatment, cardiomyocytes were transduced with adenovirus encoding PKM2 (Ad-PKM2). β-gal-overexpressing (Ad-β-gal) cells were used as controls. **(A)** Cell viability was measured in vitro via CCK-8 assay. **(B, C)** TUNEL staining was applied to analyze the number of apoptotic HL-1 cells in vitro in the presence of LPS. **(D)** ELISA-based measurement of cardiac caspase-3 activity following SC induction in vivo. **(E, F)** Results of TUNEL assays conducted in heart tissues following SC induction in vivo. **(G-L)** Analysis of contraction and relaxation parameters in field-stimulated single adult cardiomyocytes isolated from WT and PKM2^*Tg*^ mice. Values are presented as mean ± SEM. For in vivo data, *n* = 6 mice per group. For in vitro data, *n* = 4 independent experiments. #*p* < 0.05, and ##*p* < 0.01

Cardiomyocyte dysfunction and death are central events contributing to decreased myocardial function during SC. To assess whether enhanced PKM2 expression promotes cardiomyocyte viability after LPS exposure, mouse cardiac muscle HL-1 cells transfected with PKM2-overexpressing adenovirus (Ad-PKM2) or β-gal adenovirus (Ad-β-gal; control) were subjected to LPS to simulate SC in vitro. CCK-8 assays showed that LPS reduced cellular viability in cells transfected with Ad-β-gal rather than with Ad-PKM2 (Fig. 3A). Besides, TUNEL staining showed that LPS promoted apoptosis in Ad-β-gal-expressing cardiomyocytes, while this alteration was reversed in cells transfected with Ad-PKM2 (Fig. 3B and C). Accordingly, ELISA demonstrated that following LPS treatment cardiac caspase-3 activity was augmented in WT mice but remained at near-normal levels in PKM2^*Tg*^ mice (Fig. 3D). TUNEL assays further showed that LPS promoted extensive cardiomyocyte death in WT mice, and this effect was significantly attenuated in PKM2^*Tg*^ mice (Fig. 3E and F).

To assess whether PKM2 overexpression would also normalize cardiomyocyte function in the setting of SC, contraction/relaxation properties were measured in primary cardiomyocytes isolated from WT and PKM2^*Tg*^ mice after being subjected to electrical field stimulation. LPS treatment had no impact on resting adult cardiomyocyte length in any mice type (Fig. 3G). However, contractile parameters, including peak shortening (PS), maximal velocity of shortening (+ dL/dt), and time-to-peak shortening (TPS) (Fig. 3H and J), as well as relaxation parameters, i.e. maximal velocity of relengthening (-dL/dt) and time to 90% relengthening (TR90) (Fig. 3K and L), were impaired in LPS-treated WT cardiomyocytes. In contrast, contraction/relaxation properties were largely preserved in LPS-challenged PKM2^*Tg*^ cardiomyocytes. These data indicate that PKM2 supports cardiomyocyte viability and mechanical function in the setting of SC.

### PKM2 protects mitochondrial function in LPS-challenged cardiomyocytes

**Fig. 4:**
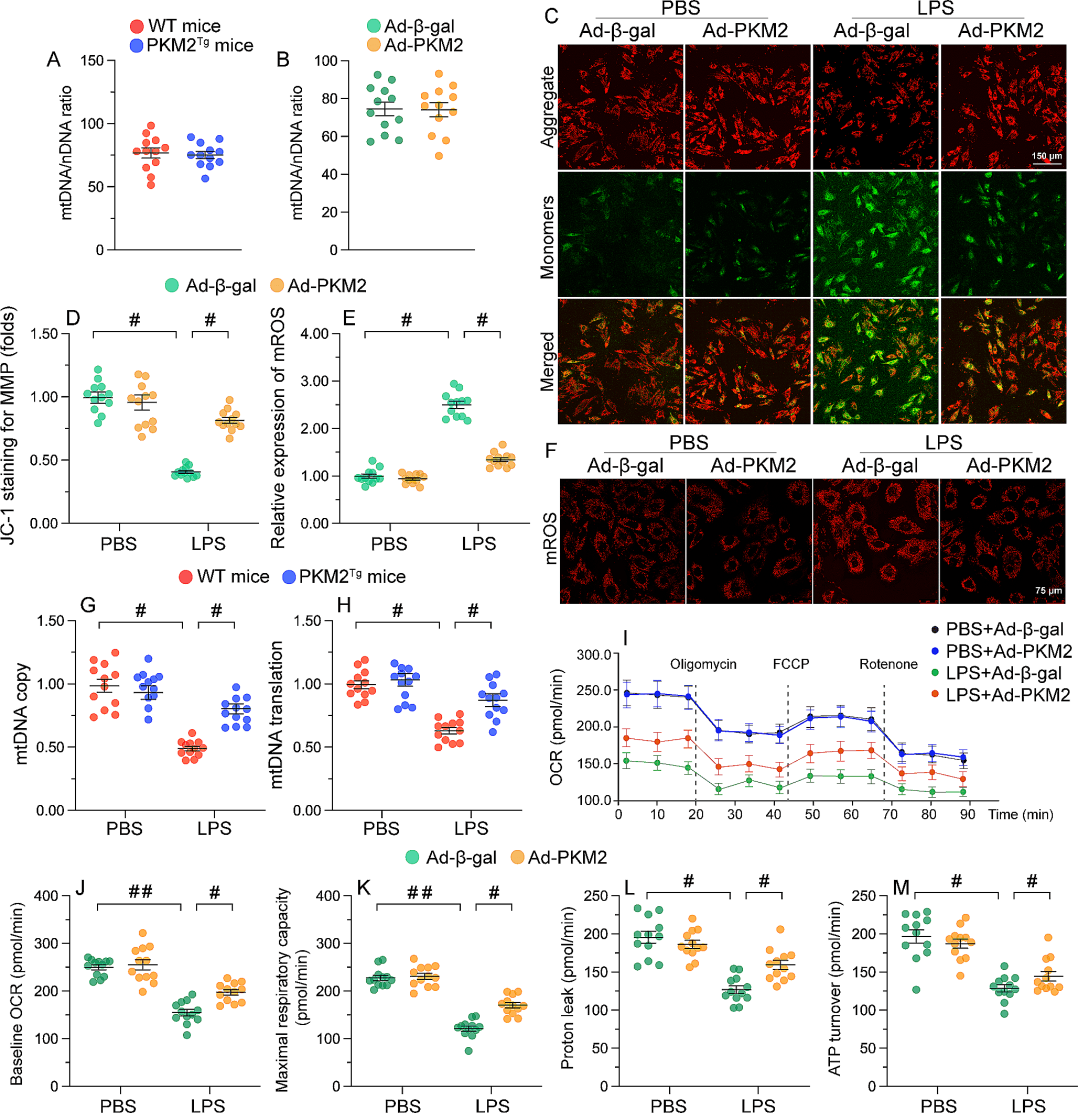
PKM2 protects mitochondrial function in cardiomyocytes exposed to LPS. PKM2 transgenic (PKM2^*Tg*^) mice and wild-type (WT) mice aged 8–10 weeks were injected intraperitoneally with 10 mg/kg LPS to induce SC. In vivo measurements were performed after 24 h later, and mice administered an equal volume of phosphate buffer saline served as controls. Immortalized mouse cardiac muscle HL-1 cells were treated with 10 µg/mL of LPS for 24 h to simulate SC in vitro. Cells treated with an equal volume of phosphate buffer saline were used as controls. Before LPS treatment, cardiomyocytes were transduced with Adenovirus encoding PKM2 (Ad-PKM2). β-gal-overexpressing (Ad-β-gal) cells were used as controls. **(A)** The *CO1* gene of mtDNA and the *NDUFV1* gene of nDNA were amplified using qPCR to assess the relative ratio of mtDNA/nDNA in heart tissues obtained from WT or PKM2^*Tg*^ mice under normal physiological conditions. **(B)** HL-1 cells were transfected with Ad-PKM2 or Ad-β-gal. Subsequently, the *CO1* gene of mtDNA and the *NDUFV1* gene of nDNA were amplified using qPCR to evaluate the relative mtDNA/nDNA ratio in vitro. **(C, D)** Analysis of the effect of LPS exposure on mitochondrial membrane potential in JC-1-loaded HL-1 cells overexpressing adenovirus PKM2 (Ad-PKM2) or β-gal (Ad-β-gal; control). **(E, F)** Detection of mitochondrial ROS production in HL-1 cells loaded with MitoSOX Red. **(G, H)** Estimation of mitochondrial DNA (mtDNA) copy number and transcription via qPCR. **(I-M)** Mitochondrial oxygen consumption rate (OCR) was determined by the Seahorse XF realtime ATP rate assay using an XF-24 Extracellular Flux Analyzer. ATP turnover, baseline OCR, maximal respiration capacity and proton leak were measured in HL-1 cells treated with Ad-β-gal or Ad-PKM2. Values are presented as mean ± SEM. For in vivo data, *n* = 6 mice per group. For in vitro data, *n* = 4 independent experiments. #*p* < 0.05, and ##*p* < 0.01

Mitochondrial damage critically contributes to cardiomyocyte dysfunction and death caused by SC [32]. Based on the above findings, we proceeded to investigate whether PKM2 overexpression was able to prevent or attenuate LPS-mediated mitochondrial damage in cultured cardiomyocytes. Initially, we utilized the mtDNA/nDNA ratio to assess potential changes in mitochondrial content or copy numbers in response to PKM2 overexpression both in vivo and in vitro. Notably, the mtDNA/nDNA ratio remained unchanged in PKM2^*Tg*^ mice compared to WT mice (Fig. 4A). Additionally, Ad-PKM2 transfection had no impact on the mtDNA/nDNA ratio (Fig. 4B). Further, as depicted in Fig. 4C and D, mitochondrial membrane potential was reduced in Ad-β-gal-transduced, control HL-1 cells, but remained stable in those overexpressing PKM2 after LPS exposure. Besides, mitochondrial ROS production was enhanced by LPS in control cells, whereas this effect was attenuated by Ad-PKM2 overexpression (Fig. 4E and F). Moreover, mitochondrial DNA (mtDNA) copy number (Fig. 4G) and mtDNA translation (Fig. 4H) were impaired in response to LPS in control cells, but largely preserved instead in PKM2-overexpressing cells. To further evaluate mitochondrial function in vitro, Seahorse assay was performed. Analysis of mitochondrial oxygen consumption rate (OCR) revealed that LPS treatment significantly impaired mitochondrial respiration in control cardiomyocytes, whereas PKM2-overexpressing cardiomyocytes were not affected (Fig. 4I and M). Collectively, our findings confirm that PKM2 overexpression can alleviate LPS-induced mitochondrial dysfunction in cardiac cells.

### PKM2 overexpression maintains mitochondrial quality control in cardiomyocytes challenged with LPS

**Fig. 5:**
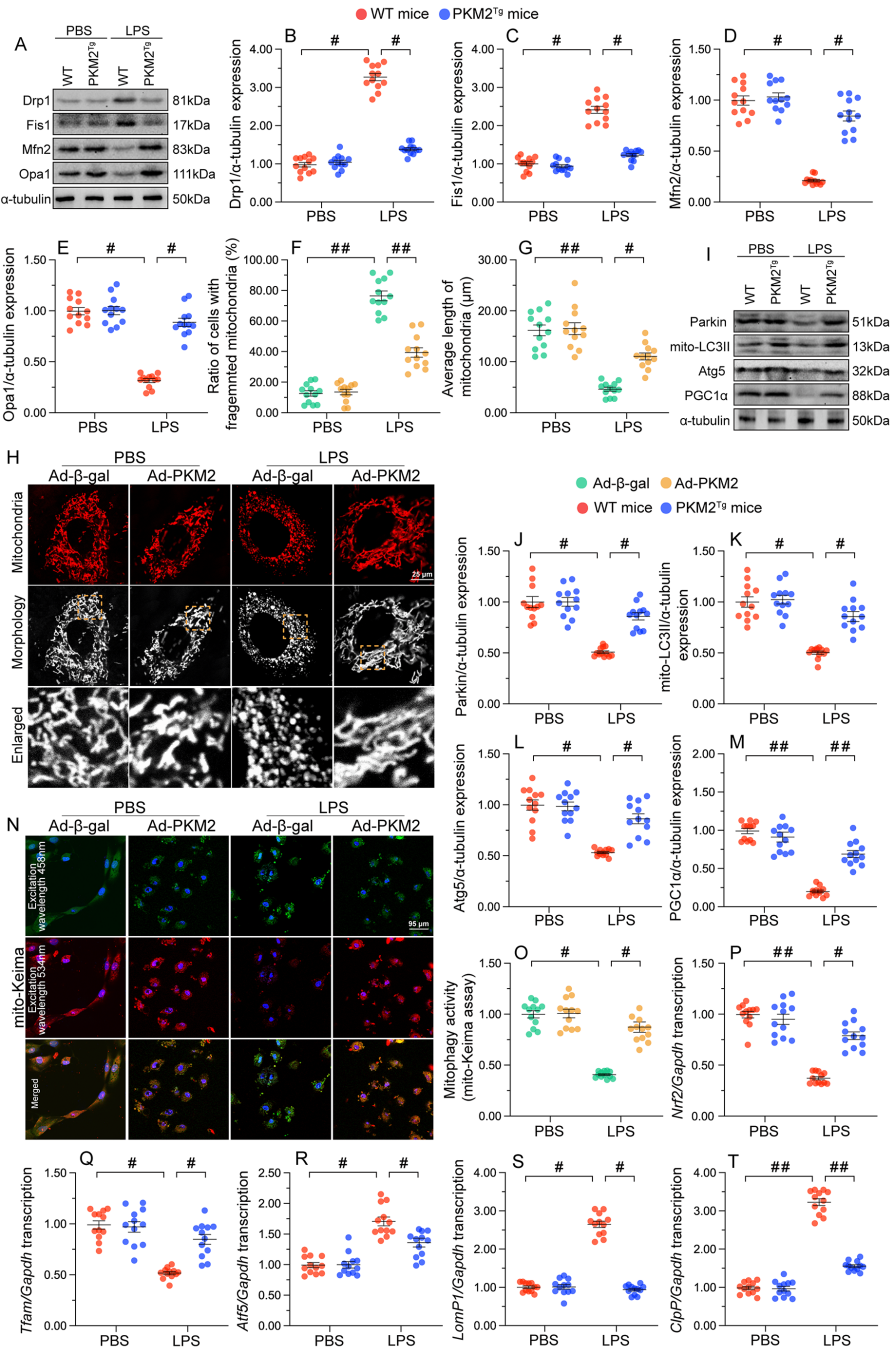
PKM2 overexpression maintains mitochondrial quality control. PKM2 transgenic (PKM2^*Tg*^) mice and wild-type (WT) mice aged 8–10 weeks were injected intraperitoneally with 10 mg/kg LPS to induce SC. In vivo measurements were performed after 24 h later, and mice administered an equal volume of phosphate buffer saline served as controls. Immortalized mouse cardiac muscle HL-1 cells were treated with 10 µg/mL of LPS for 24 h to simulate SC in vitro. Cells treated with an equal volume of phosphate buffer saline were used as controls. Before LPS treatment, cardiomyocytes were transduced with Adenovirus encoding PKM2 (Ad-PKM2). β-gal-overexpressing (Ad-β-gal) cells were used as controls. **(A-E).** Western blot analysis of cardiac Drp1, Fis1, Mfn2, and Opa1 expression in vivo. **(F-H)** Results of immunofluorescence analysis of mitochondrial morphology in HL-1 cells. Rate of cells with fragmented mitochondria (F), average length of mitochondria (G), and representative mitochondrial immunofluorescence images (H) are shown. **(I-M)** Representative immunoblots and quantification of changes in Parkin, Atg5, Beclin1, and PGC1α expression in cardiac tissues. **(N, O).** Representative images and quantitative results of mt-Keima assays assessing mitophagy in HL-1 cells. **(P-T)** Transcriptional analysis of cardiac *Nrf2*, *Tfam*, *Atf6*, *LonP1*, and *mtHsp70* expression by qPCR in vivo. Values are presented as mean ± SEM. For in vivo data, *n* = 6 mice per group. For in vitro data, *n* = 4 independent experiments. #*p* < 0.05, and ##*p* < 0.01

In response to mitochondrial damage, a series of endogenous adaptive mechanisms, including mitochondrial fission/fusion, mitophagy, organelle biogenesis, and the mitochondrial unfolded protein response (mtUPR), collectively exerting mitochondrial quality control (MQC), are engaged to preserve mitochondrial morphology and function [15, 33, 34]. To study the molecular basis underlying the protective effect of PKM2 expression on mitochondrial integrity in cardiomyocytes challenged with LPS, we investigated whether MQC was governed and/or normalized by PKM2. Quantitative protein analysis showed that LPS exposure upregulated levels of Drp1 and Fis1 and suppressed the expression of Mfn2 and Opa1 (Fig. 5A and E), indicative of increased fission and decreased fusion in WT mouse heart tissues. Whereas these changes were negated by PKM2 overexpression. In vitro immunofluorescence analysis of HL-1 cardiomyocyte mitochondria showed that the mitochondrial network was disrupted in the presence of LPS (Fig. 5F and H), with a significant drop in average mitochondrial length and an elevation in the proportion of cardiomyocytes with fragmented mitochondria. In contrast, mitochondrial morphology was preserved in cardiomyocytes overexpressing PKM2 (Fig. 5F and H).

Regarding mitophagy and mitochondrial biogenesis, western blot analysis revealed that cardiac levels of Parkin, Atg5, Beclin1, and PGC1α were significantly downregulated in WT mice after LPS treatment. In contrast, PKM2^*Tg*^ mice exhibited near-normal levels of these proteins (Fig. 5I and M). Mt-Keima assays, used to monitor mitophagy activity, showed in turn that mitophagic flux was impaired by LPS in control HL-1 cells and restored after PKM2 overexpression (Fig. 5N and O). In addition, the transcriptions of *Nrf2* and *Tfam*, two downstream effectors of PGC1α-mediated mitochondrial biogenesis, were blunted by LPS in WT mice but restored upon PKM2 overexpression (Fig. 5P and Q). The mtUPR, encompasses an adaptive transcriptional stress program, activated by disrupted mitochondrial proteostasis, that mediates the expression of genes in response to stress [35]. We noted that after SC induction, cardiac transcriptions of *Atf6*, *LonP1*, and *mtHsp70* were upregulated in WT mice but maintained to low levels in PKM2^*Tg*^ mice comparable with untreated WT mice (Fig. 5R and T). Thus, the above data showed that PKM2 overexpression maintains MQC function in cardiac cells exposed to LPS.

### PKM2 binds to and prevents PHB2 degradation

**Fig. 6:**
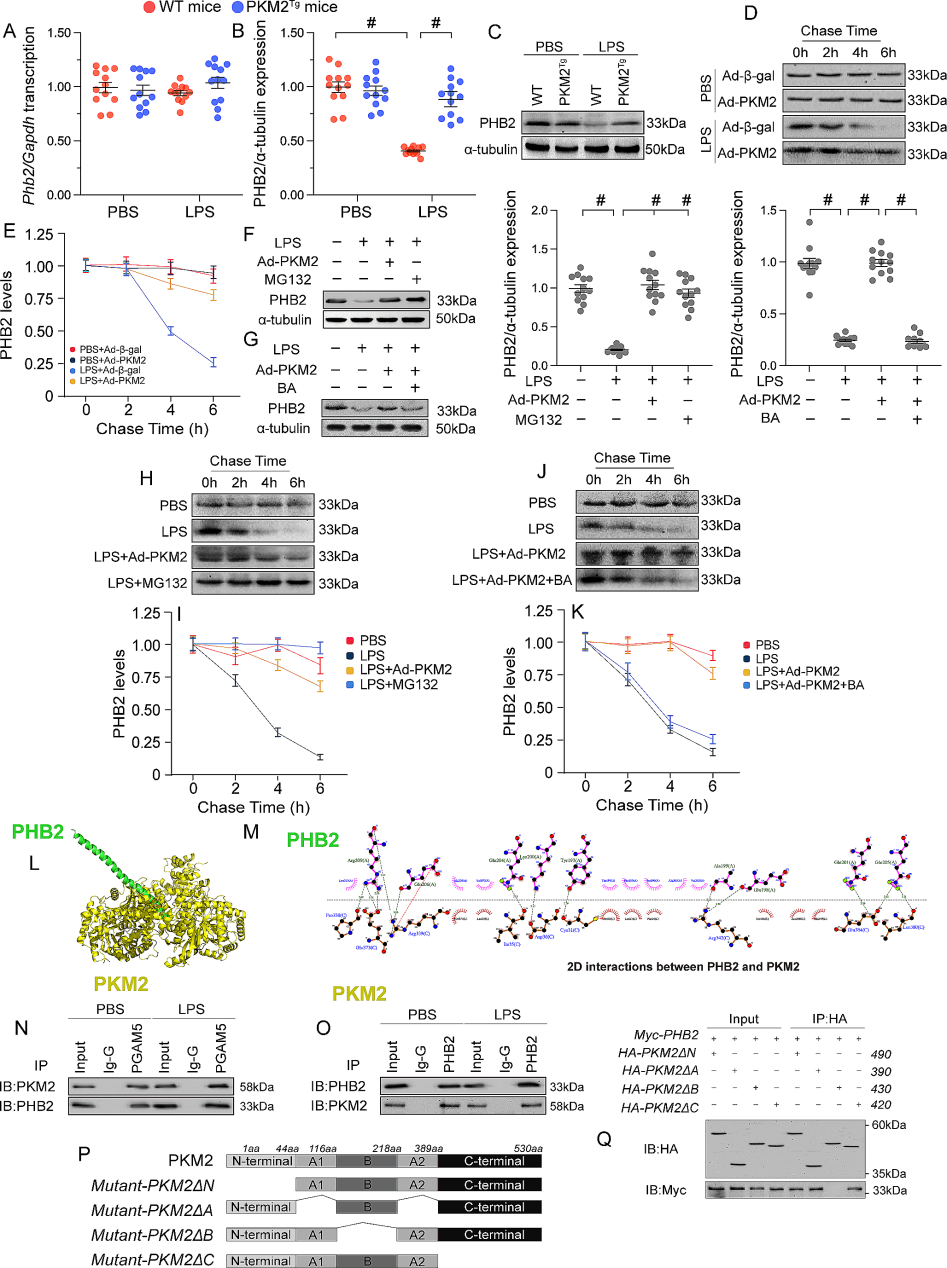
PKM2 binds to and prevents PHB2 degradation. PKM2 transgenic (PKM2^*Tg*^) mice and wild-type (WT) mice aged 8–10 weeks were injected intraperitoneally with 10 mg/kg LPS to induce SC. In vivo measurements were performed after 24 h later, and mice administered an equal volume of phosphate buffer saline served as controls. Immortalized mouse cardiac muscle HL-1 cells were treated with 10 µg/mL of LPS for 24 h to simulate SC in vitro. Cells treated with an equal volume of phosphate buffer saline were used as controls. Before LPS treatment, cardiomyocytes were transduced with Adenovirus encoding PKM2 (Ad-PKM2). β-gal-overexpressing (Ad-β-gal) cells were used as controls. **(A)** Analysis of the effect of LPS exposure on cardiac *Phb2* mRNA expression levels in WT and PKM2^*Tg*^ mice. **(B, C)** Western blots analysis of cardiac PHB2 protein levels in WT and PKM2^*Tg*^ mice. **(D, E)** Analysis of the half-life of PHB2 protein in HL-1 cells (pulse-chase assay). **(F-G)** Western blot analysis of PHB2 expression in HL-1 cells exposed to LPS in the presence or absence of MG132, a proteasome inhibitor, or betulinic acid (BA), a proteasome activator. **(H, I)** Pulse-chase analysis of PHB2 degradation rate in cultured in HL-1 cells treated with LPS and MG132. **(J, K)** Pulse-chase analysis of PHB2 degradation rate in cultured in HL-1 cells treated with LPS and BA. **(L-M)** Mapping of PHB2/PKM2 interacting regions by docking analysis. **(N, O)** Co-IP analysis of PKM2/PHB2 binding in HL-1 cells transfected with different domain deletion PKM2 mutants. **(P)** Mapping of PKM2 regions and deletion mutants. **(Q)** Western blot analysis of PHB2 expression in HL-1 cells transfected with domain deletion PKM2 mutants. Values are presented as mean ± SEM. For in vivo data, *n* = 6 mice per group. For in vitro data, *n* = 4 independent experiments. #*p* < 0.05

Recent research has identified PHB2 as a regulator of MQC [36]. To discern the molecular mechanism by which PKM2 preserves MQC in LPS-induced SC, we focused on potential alterations in PHB2 expression. qPCR analysis showed that neither LPS nor PKM2 overexpression influenced cardiac PHB2 mRNA expression (Fig. 6A). However, western blot assays showed that after LPS administration cardiac PHB2 expression was downregulated in WT mice and sustained at near-baseline levels in PKM2^*Tg*^ mice (Fig. 6B and C). The correlation between PHB2 and PKM2 expression led us to speculate that the post- translational stability of PHB2 may be under the control of PKM2. Pulse-chase analysis on HL-1 cells further showed that LPS increased the degradation rate of endogenous PHB2 protein in control cells, and this effect was prevented by PKM2 overexpression (Fig. 6D and E).

In line with these findings, LPS-mediated PHB2 downregulation was nullified upon incubation of HL-1 cardiomyocytes with the proteasome inhibitor MG132 (Fig. 6F-G), suggesting involvement of the ubiquitin-proteasome system. On the contrary, and confirming proteasome involvement, PKM2 overexpression failed to sustain PHB2 expression in LPS-treated cardiomyocytes pre-incubated with betulinic acid (BA), a potent proteasome activator (Fig. 6H-I). Accordingly, protein stability analysis using pulse-chase assays showed that MG132 exposure counteracted LPS-stimulated PHB2 degradation (Fig. 6J), whereas PKM2 overexpression failed to extend the half-life of PHB2 in BA-treated cardiomyocytes (Fig. 6K). These results suggested that PKM2 overexpression in cardiomyocytes attenuates proteasome-mediated PHB2 degradation.

To elucidate the molecular basis underlying PKM2-mediated PHB2 stability, we evaluated the presence of constitutive interactions between PKM2 and PHB2 in cultured HL-1 cells. First, the active regions of PKM2 and PHB2 were screened by molecular docking simulations. We detected the existence of H-bond or hydrophobic interactions between PKM2 and PHB2 (Fig. 6L and M), with a minimum binding energy of -12.3 kcal·mol^− 1^. Co-IP assays further confirmed an endogenous interaction between PKM2 and PHB2 under physiological condition or LPS stress (Fig. 6N and O). To define the structural moieties required for PKM2/PHB2 binding, we first mapped the regions of PKM2 that are required for protein-protein binding. Since PKM2 has four domains, we generated four different domain-deletion PKM2 mutants (Fig. 6P) and transfected them into HL-1 cardiomyocytes. Co-IP assays showed that PKM2ΔA, PKM2ΔC, and PKM2ΔN, but not PKM2ΔB, were able to pull-down PHB2 (Fig. 6Q).

### PKM2 induces PHB2 phosphorylation

**Fig. 7:**
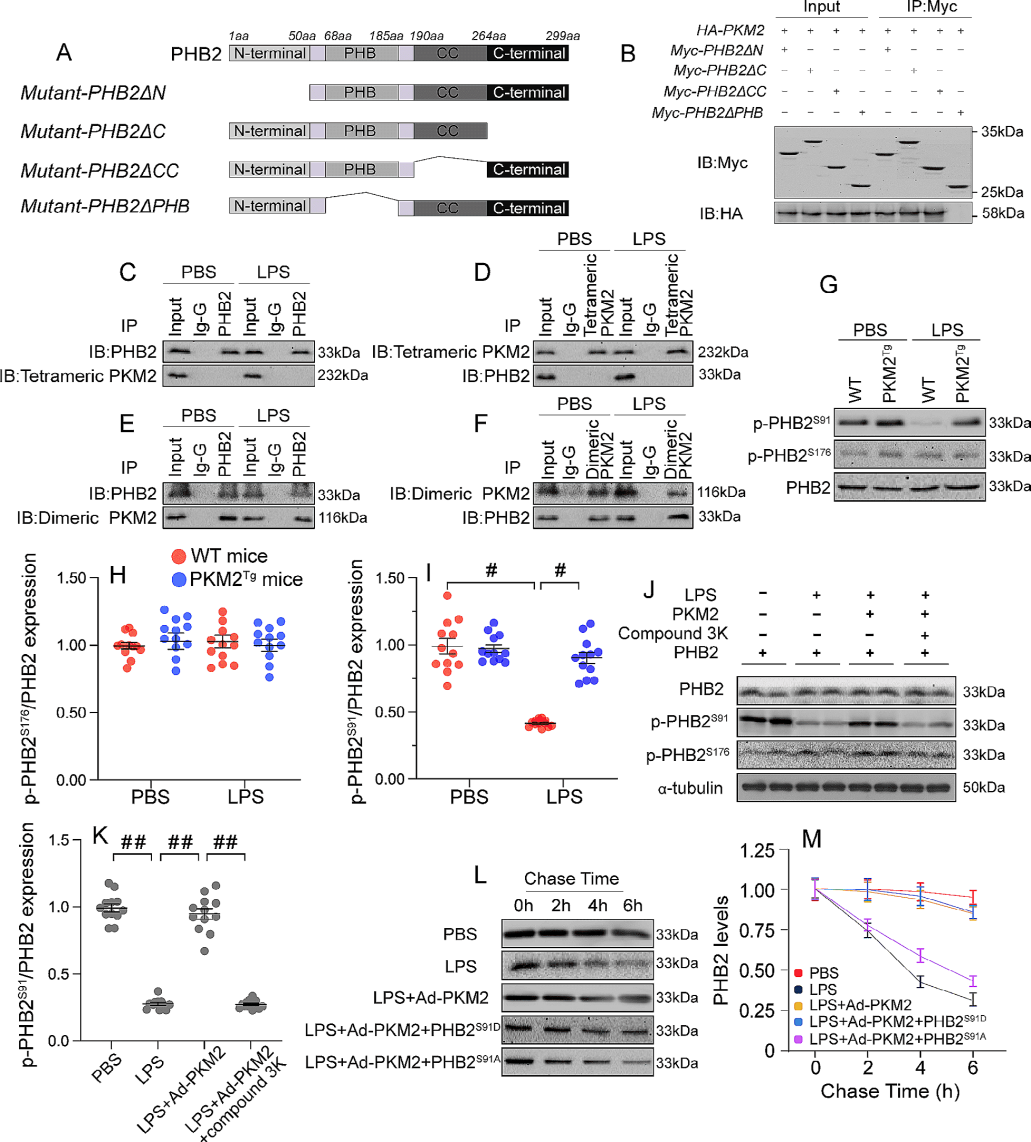
PKM2 induces PHB2 phosphorylation. PKM2 transgenic (PKM2^*Tg*^) mice and wild-type (WT) mice aged 8–10 weeks were injected intraperitoneally with 10 mg/kg LPS to induce SC. In vivo measurements were performed after 24 h later, and mice administered an equal volume of phosphate buffer saline served as controls. Immortalized mouse cardiac muscle HL-1 cells were treated with 10 µg/mL of LPS for 24 h to simulate SC in vitro. Cells treated with an equal volume of phosphate buffer saline were used as controls. Before LPS treatment, cardiomyocytes were transduced with Adenovirus encoding PKM2 (Ad-PKM2). β-gal-overexpressing (Ad-β-gal) cells were used as controls. **(A)** Mapping of PHB2 regions and deletion mutants. **(B)** Representative immunoblot from immunoprecipitation analysis of HL-1 cells transfected with PHB2 deletion mutants. **(C-F)** Co-IP analysis of the interaction between dimeric and tetrameric PKM2 forms and PHB2 in HL-1 cells. **(G-I)** Western blot analysis of p-PHB2^S176^ and p-PHB2^Ser91^ expression in cardiac tissues. **(J, K)** Representative results of an in vitro kinase assay evaluating binding of phospho-PHB2 variants to PKM2 in the presence of LPS or compound 3k, a PKM2 inhibitor. **(L, M)** Pulse-chase analysis of PHB2 protein half-life in HL-1 cells transfected with PHB2^S91D^ and PHB2^S91A^ mutant constructs. Values are presented as mean ± SEM. For in vivo data, *n* = 6 mice per group. For in vitro data, *n* = 4 independent experiments. #*p* < 0.05, and ##*p* < 0.01

After we revealed an interaction between PKM2 and PHB2, we asked whether conformational changes occur in PHB2 for its stabilization after PKM2 binding. To address this question, we analyzed the regions of PHB2 that are required for its interaction with PKM2. As PHB2 has four major domains, we generated four different domain deletion PHB2 mutants (Fig. 7A) and transfected them into HL-1 cardiomyocytes. Co-IP assays showed that PHB2ΔN, PHB2ΔCC, and PHB2ΔC, but not PHB2ΔPHB, were able to pull-down PKM2 (Fig. 7B). This suggests that the PHB domain of PHB2 determines its binding to PKM2.

PKM2 has two conformations, a tetrameric one with high protein kinase (PK) activity that catalyzes the production of pyruvate from phosphoenolpyruvate (PEP), and a dimeric one, which has lower PK activity and also exerts non-glycolytic enzymatic functions. Interestingly, co-IP results showed that PHB2 mainly interacted with dimeric, rather than tetrameric, PKM2 (Fig. 7C and F). Thus, LPS seems to interrupt the cooperation between PHB2 and dimeric PKM2. These results hinted that the kinase function of PKM2 is involved in the regulation of the PHB domain of PHB2. PKM2 has been reported to directly induce post-transcriptional phosphorylation of several proteins, such as ERK2^T202^ [37], β-catenin^Y333^ [38], histone H3^T11^ [39], myosin light chain 2^Y118^ [40], and protein kinase B substrate l (AKT1S1)^S202^ [41]. On the other hand, PHB2 possesses three phosphorylation sites (S91, S176, and S243) which have been reported to enhance the anti-oxidative and anti-apoptotic properties of PHB2 through an undefined mechanism [42–44]. Since the PHB domain of PHB2 encompasses amino acids 68–185, we asked whether PKM2/PHB2 interaction results in PHB2 phosphorylation within this sequence fragment. Western blotting showed abundant expression of p-PHB2^S91^ and little p-PHB2^S176^ in heart tissues under baseline conditions. Neither LPS nor PKM2 overexpression had an impact on p-PHB2^S176^ expression (Fig. 7G and I). In contrast, after exposure to LPS the expression of p-PHB2^S91^ was downregulated in WT mice but remained at near normal levels in PKM2^*Tg*^ mice, compared with PBS treated mice (Fig. 7G and I). An in vitro kinase assay further showed that PKM2 induced PHB2 phosphorylation at S91, and this alteration was significantly inhibited by compound 3k, a PKM2 inhibitor (Fig. 7J and K).

To evaluate whether PKM2-mediated PHB2 phosphorylation at S91 is necessary for PHB2 stabilization, phosphorylation-defective (PHB2^S91A^) and phosphorylation-mimetic (PHB2^S91D^) PHB2 mutants were transfected into HL-1 cells. After transfection with PHB2^S91D^, LPS-mediated PHB2 degradation was delayed in control cells (Fig. 7L and M). In turn, in cells transfected with a PHB2^S91A^ mutant, PKM2 overexpression failed to maintain PHB2 stability (Fig. 7L and M). Taken together, our results showed that PKM2 binds to PHB2 and phosphorylates it at S91, leading to increased PHB2 stability.

### PHB2 dephosphorylation abolishes PKM2-mediated mitochondrial protection

**Fig. 8:**
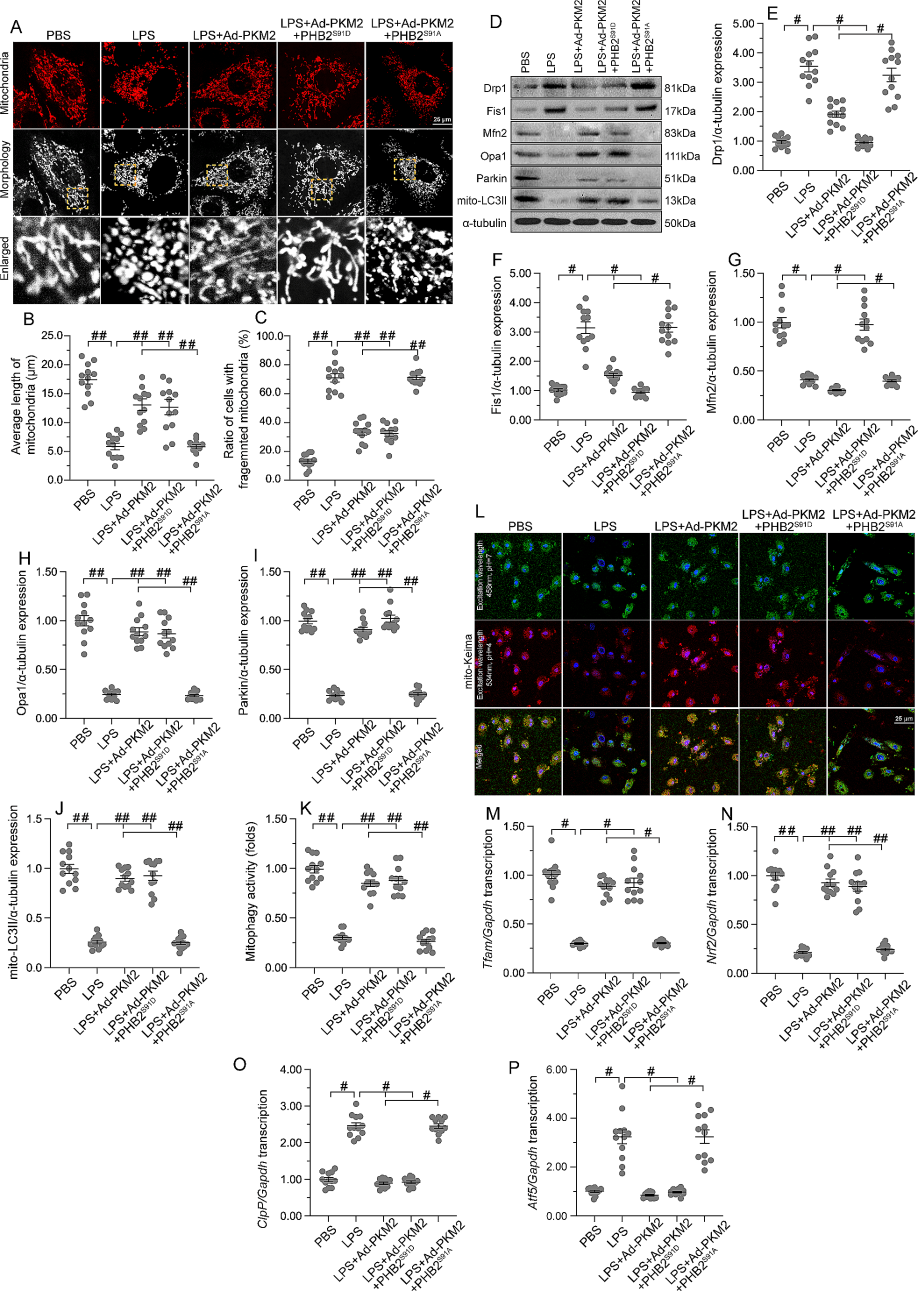
PHB2 dephosphorylation abolishes PKM2-mediated mitochondrial protection. HL-1 cardiomyocytes were transfected with PKM2 overexpression adenovirus (Ad-PKM2), a phosphorylation-defective PHB2^S91A^ mutant, or a phosphorylation-mimetic PHB2 ^S91D^ mutant before LPS treatment. Adenovirus loaded β-gal (Ad-β-gal) cells were used as controls. **(A-C)** Immunofluorescence analysis of mitochondrial morphology in HL-1 cells. Representative images of mitochondria immunofluorescence (A), average mitochondrial length (B), and proportion of cardiomyocytes with fragmented mitochondria (C) are shown. **(D-J)** Western blot analysis of Drp1, Fis1, Mfn2, Opa1, Parkin, and Atg5 in HL-1 cells. **(K, L)** Mitophagy analysis results (mt-Keima assay). **(M-P).** Transcriptional analysis of *Tfam*, *Nrf2*, *mtHsp70*, and *Atf6* expression by qPCR. Values are presented as mean ± SEM. For in vivo data, *n* = 6 mice per group. For in vitro data, *n* = 4 independent experiments. #*p* < 0.05, and ##*p* < 0.01

To evaluate whether PHB2 phosphorylation underlies PKM2-related MQC activation during SC, further assays were conducted in HL-1 cells transfected with PHB2^S91A^ and PHB2^S91D^ mutants. Immunofluorescent assessment of mitochondrial morphology showed that LPS-induced mitochondrial fragmentation was attenuated by overexpression of either PHB2^S91D^ or PKM2 (Fig. 8A and C). However, in HL-1 cells transfected with PHB2^S91A^, PKM2 overexpression failed to maintain mitochondrial length and reduce the number of cardiomyocytes with plenty of fragmented mitochondria (Fig. 8A and C). Further, western blot analysis showed that LPS-mediated disruption of mitochondrial fission/fusion and mitophagy could be normalized by overexpression of PHB2^S91D^ or PKM2 (Fig. 8D and J). In contrast, in HL-1 cells transfected with PHB2^S91A^, PKM2 overexpression failed to sustain mitochondrial fission/fusion and mitophagy (Fig. 8D and J). Similarly, mt-Keima assays confirmed that either PHB2^S91D^ or PKM2 overexpression was able to sustain mitophagy in the presence of LPS, while PKM2-induced mitophagy was nullified by PHB2^S91A^ transfection (Fig. 8K and L). Furthermore, mitochondrial biogenesis was stimulated by PHB2^S91D^ or PKM2 overexpression in LPS-treated cells, while the effect of PKM2 overexpression was abrogated by PHB2^S91A^ transfection (Fig. 8M and N). Lastly, transcriptions of mtUPR-related genes were slightly increased by LPS and partially restored toward baseline levels upon overexpression of either PHB2^S91D^ or PKM2 (Fig. 8O-P). However, in cells transfected with the PHB2^S91A^ mutant, the inhibitory effect of PKM2 on mtUPR was inhibited (Fig. 8O and P). These data indicate that PKM2-mediated mitochondrial protection occurs through PKM2-dependent PHB2 phosphorylation.

### PKM2-mediated cardiomyocyte protection against septic insult requires PHB2 phosphorylation

**Fig. 9:**
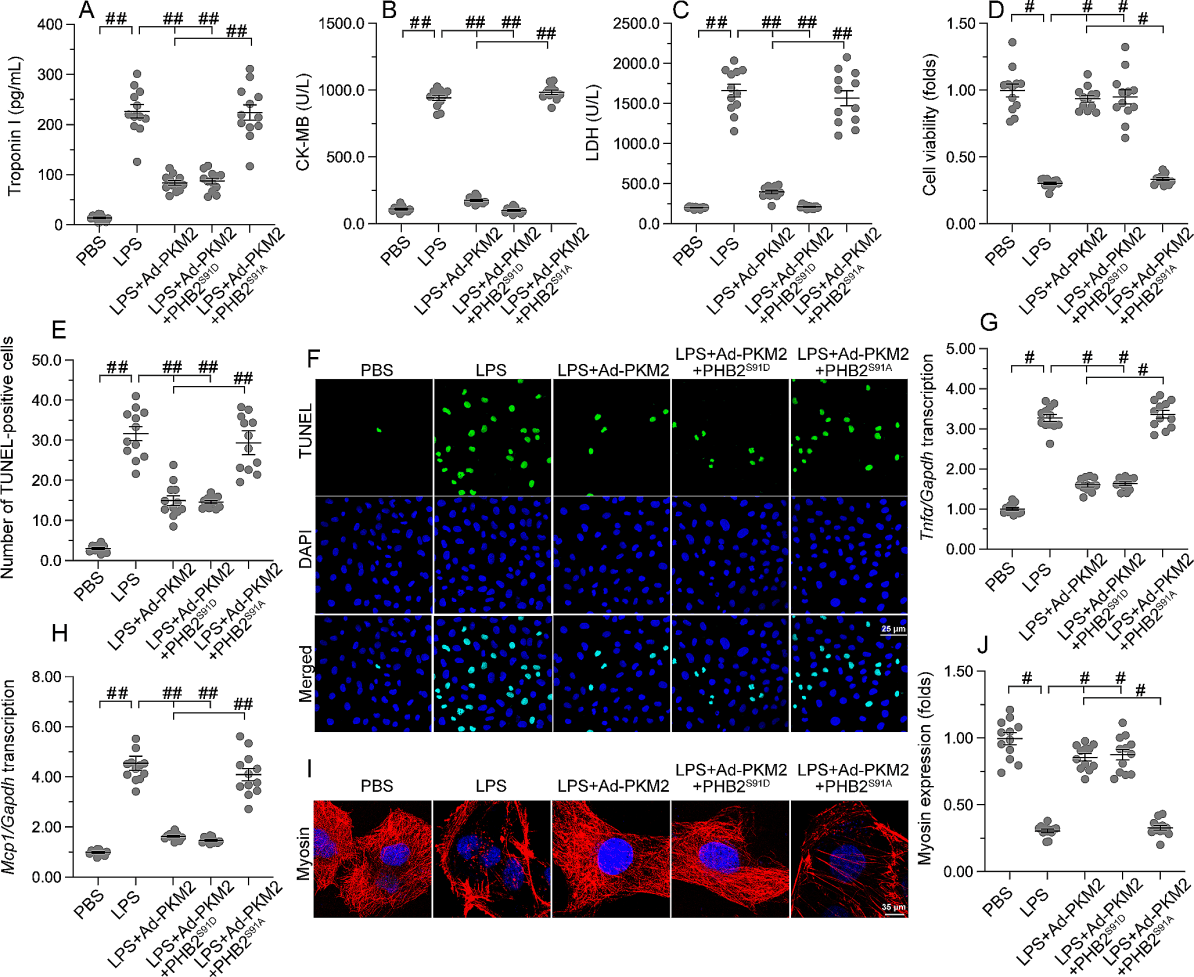
PKM2-mediated cardiomyocyte protection against septic insult requires PHB2 phosphorylation. HL-1 cardiomyocytes were transfected with PKM2 overexpression Adenovirus (Ad-PKM2), a phosphorylation-defective PHB2^S91A^ mutant, or a phosphorylation-mimetic PHB2 ^S91D^ mutant before LPS treatment. Adenovirus loaded β-gal (Ad-β-gal) cells were used as controls. **(A, C)** ELISA-based analysis of TnI, CK-MB, and LDH levels in culture media of HL-1 cells transfected with phosphorylation-defective (PHB2^S91A^) and phosphorylation-mimetic (PHB2^S91D^) mutant constructs. **(D)** Cell viability analysis via CCK-8 assay in vitro. **(E, F)** Apoptosis analysis by TUNEL staining in cultured HL-1 cells. **(G, H)** Transcriptional analysis of *Tnfα* and *Mcp1* expression. **(I, J)** Representative images of myosin immunofluorescence. Myosin expression levels were normalized to those of the control group. Values are presented as mean ± SEM. For in vivo data, *n* = 6 mice per group. For in vitro data, *n* = 4 independent experiments. #*p* < 0.05, and ##*p* < 0.01

To illustrate the necessary role of PHB2 phosphorylation in supporting PKM2-induced cardioprotection, HL-1 cardiomyocyte viability and function were determined after transfection with PHB2^S91D^ or PHB2^S91A^ mutants. LPS exposure promoted the release of cardiac injury markers, e.g. TnI, CK-MB, and LDH, into the culture medium in control cells, and this trend was decreased by either PKM2 overexpression or introduction of the phospho-mimetic PHB2^S91D^ mutant (Fig. 9A and C). In line with the findings described above, PKM2 overexpression failed to prevent the upregulation of cardiac damage markers in HL-1 cells co-transfected with the phospho-defective PHB2^S91A^ mutant after LPS challenge (Fig. 9A and C). In turn, both cell viability and survival assessed by CCK-8 assay (Fig. 9D) and TUNEL staining (Fig. 9E and F), respectively, were improved by either PKM2 overexpression or PHB2^S91D^ mutant transfection. In contrast, PKM2 overexpression-mediated cardiomyocyte survival was decreased in cells co-expressing PHB2^S91A^ (Fig. 9D and F). Moreover, LPS-stimulated transcription of pro-inflammatory factors was significantly suppressed by either PKM2 or PHB2^S91D^ overexpression, while the anti-inflammatory action of PKM2 was impaired upon transfection of PHB2^S91A^ (Fig. 9G-H). Finally, either PKM2 or PHB2^S91D^, but not PHB2^S91D^ transfection, largely preserved myosin filament expression and organization in cardiomyocytes exposed to LPS (Fig. 9I-J). The above evidence thus confirms that PKM2-mediated cardioprotection during septic conditions requires PHB2 phosphorylation.

### Knockin mice expressing phospho-mimetic *Phb2*^S91D^ are less vulnerable to SC

**Fig. 10:**
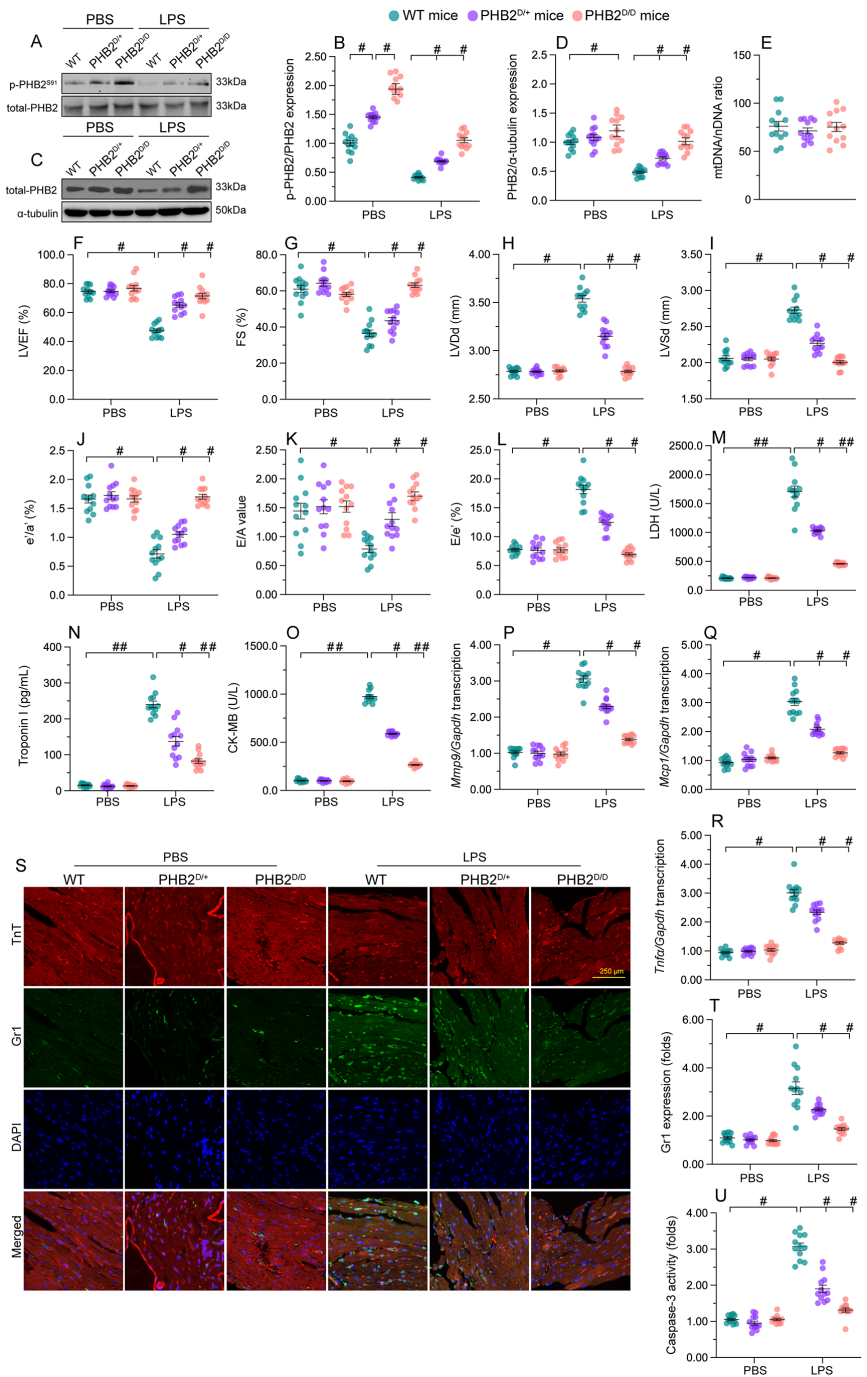
Knockin mice expressing phospho-mimetic Phb2^S91^ are less vulnerable to SC. Heterozygous *Phb2S91*^D/+^ mice, homozygous *Phb2S91*^*D/D*^ mice, and wild-type (WT) mice aged 8–10 weeks were injected intraperitoneally with 10 mg/kg LPS to induce SC. Mice administered an equal volume of phosphate buffer saline served as controls. **(A, B)** Western blot analysis of cardiac p-PHB2^S91^ in WT, heterozygous *Phb2S91*^D/+^, and homozygous *Phb2S91*^*D/D*^ mice treated with PBS or LPS. Total-PHB2 was used as the loading control. **(C, D)** Western blot analysis of total-PHB2 in WT, heterozygous *Phb2S91*^D/+^, and homozygous *Phb2S91*^*D/D*^ mice treated with PBS or LPS. α-tubulin was used as the loading control. **(E)** The *CO1* gene of mtDNA and the *NDUFV1* gene of nDNA were amplified using qPCR to assess the relative ratio of mtDNA/nDNA in heart tissues obtained from WT, heterozygous *Phb2S91*^D/+^, and homozygous *Phb2S91*^*D/D*^ mice under normal physiological conditions. **(F-L)** Echocardiographic evaluation. LVDd, left ventricular diastolic dimension; LVDs, left ventricular systolic dimension; IVS, interventricular septum thickness; E/A, ratio of early to late transmitral flow velocities; FS, ratio of left ventricular fractional shortening. **(M-O)** ELISA-based analysis of serum TnI, CK-MB, and LDH concentrations. **(P-R)** Transcriptional analysis of cardiac *Mmp9*, *Mcp1*, and *Tnfα* expression by qPCR. **(S)** Representative images of TnT and Gr-1 immunohistochemistry in cardiac sections. (**T)** Quantification of Gr-1-positive neutrophils in mouse heart tissues. **(U)** ELISA-based measurement of caspase-3 activity in heart tissues. Values are presented as mean ± SEM. For in vivo data, *n* = 6 mice per group. For in vitro data, *n* = 4 independent experiments. #*p* < 0.05, and ##*p* < 0.01

To investigate whether PKM2-mediated PHB2 phosphorylation confers myocardial protection against SC in vivo, transgenic knockin mice carrying the *Phb2*^*S91D*^ variant were generated. Total-PHB2 was used as the loading control to analyze the changes in p-PHB2^S91^. In comparison to WT mice, heart tissues from homozygous *Phb2S91*^*D/D*^ mice showed elevated levels of p-PHB2^S91^ (Fig. 10A-B). Since PHB2 phosphorylation prevents PHB2 degradation, we further examined the alterations in total-PHB2 expression in WT, heterozygous *Phb2S91*^D/+^, and homozygous *Phb2S91*^*D/D*^ mice. After normalizing to α-tubulin, the levels of total-PHB2 were significantly reduced in LPS-treated WT mice (Fig. 10C-D). However, these changes were partially prevented in *Phb2S91*^D/+^ mice and fully restored in *Phb2S91*^*D/D*^. Furthermore, the mtDNA/nDNA ratio was employed to assess potential alterations in mitochondrial content or mitochondrial copy numbers in transgenic knockin mice harboring the *Phb2*^*S91D*^ variant. As depicted in Fig. 10E, the mtDNA/nDNA ratio exhibited no significant changes among WT, heterozygous *Phb2S91*^*D/+*^, and homozygous *Phb2S91*^*D/D*^ mice.

Heart function, assessed by echocardiography, was slightly improved in *Phb2S91*^*D/+*^ mice (Fig. 10F and L), and largely normalized in *Phb2S91*^*D/D*^ mice upon LPS-induced SC. Similarly, LPS-stimulated TnI, CK-MB, and LDH were partially reduced in *Phb2S91*^*D/+*^ mice and significantly inhibited in *Phb2S91*^*D/D*^ mice (Fig. 10M and O). Along with these changes, LPS-induced transcription of *Mmp9*, *Mcp1*, and *Tnfα* was downregulated in *Phb2S91*^*D/+*^ mice, and further repressed in *Phb2S91*^*D/D*^ mice (Fig. 10P and R). In accordance with these alterations, myocardial accumulation of Gr-1 positive neutrophils was relived in *Phb2S91*^*D/+*^ mice and completely prevented in *Phb2S91*^*D/D*^ mice (Fig. 10S and T). Lastly, LPS-induced cardiomyocyte death, as assessed by caspase-3 activity, was partly attenuated in *Phb2S91*^*D/+*^ mice and remarkably inhibited in *Phb2S91*^*D/D*^ mice (Fig. 10U). Taken together, our results elucidated that PHB2 phosphorylation mediates myocardial protection against LPS-induced SC.

## Discussion

The present study focused on the role of PKM2 in cardiomyocyte homeostasis and MQC regulation in the setting of SC. Our main findings showed that: (1) LPS reduced the levels of myocardial PKM2 mainly through suppressed expression of dimeric PKM2; (2) PKM2 overexpression in mice correlated with reduced myocardial inflammation and cardiomyocyte death after SC induction; (3) overexpression of PKM2 improved heart performance through repressing LPS-triggered mitochondrial damage; (4) PKM2-mediated mitochondrial protection in the presence of LPS correlates with normalized MQC, as evidenced by balanced mitochondrial fission/fusion events, activated mitophagy, increased mitochondrial biogenesis, and mitigated mtUPR; (5) upon LPS stress, PKM2 stimulated MQC through sustaining PHB2 expression; (6) PKM2 interacts directly with PHB2 and induces PHB2 phosphorylation at Ser91, resulting in increased PHB2 stability; (7) the protective actions of PKM2 on cardiomyocyte homeostasis and mitochondrial integrity are nullified upon expression of a phosphorylation-defective PHB2^S91A^ mutant construct. This study thus identified PHB2 dephosphorylation, leading to enhanced PHB2 protein degradation, as an initial signal determining MQC disruption during development of SC. Besides, we also showed that stabilized PKM2 expression is a prerequisite to sustain PHB2 phosphorylation after septic insult. These findings indicate that PKM2 downregulation, PKM2-PHB2 disassociation, PHB2 dephosphorylation, and MQC dysfunction are important determinators of sepsis-triggered myocardial injury.

Increasing evidence supports the beneficial effects offered by PKM2 in cardiovascular disorders. During cardiac development, loss of PKM2 in cardiomyocytes impairs cell cycling and reduces cardiomyocyte numbers and myocardium size [30]. By comparison, overexpression of PKM2 in heart tissues via cardiomyocyte-specific *Pkm2* modified RNA protected the myocardium against acute or chronic myocardial infarction through enhancing heart function, inhibiting cell death, and repressing oxidative stress [30]. Mechanistically, PKM2-mediated cardioprotection seems to correlate with activation of anabolic pathways and β-catenin signaling, suggesting the dimeric PKM2, rather than tetrameric PKM2, is essential for maintaining heart performance [30]. Apart from its actions on cardiomyocytes, pharmacological activation of PKM2 in endothelial cells promotes proliferation and induces angiogenic differentiation [26]. Instead of mediating metabolic adaption, this beneficial effect has been reported to be associated with increased mitochondrial fusion/fission ratio, an effect accompanied by stabilized mitochondrial potential, decreased mitochondrial ROS generation, and angiogenesis-related gene expression [26]. Importantly, pulmonary hypertension [45], angiotensin II-triggered cardiac remodeling [46], and diabetic cardiomyopathy [47] were all shown to be relieved by PKM2 activation, either by genetic modification or pharmacological manipulation, through multiple mechanisms such as inhibition of TGF-β/Smad2/3 and Jak2/Stat3 pathways, oxidative stress, and NLRP3 inflammasome. These results highlight an unforeseen non-metabolic regulatory effect of PKM2 in cardioprotection. In accordance with the above results, our data also showed that SC is featured by a drop in cardiac levels of dimeric PKM2 and that re-introduction of PKM2 improves heart function through sustaining MQC. However, strategies aimed at modulating PKM2 expression require cautiousness, as the impact of its tetrameric and dimeric forms on heart performance would be different. Tetrameric PKM2 would increase glycolysis, a process less efficient than mitochondrial respiration in terms of ATP yield per glucose, despite demanding lower oxygen consumption. Of note, elevated PKM2-mediated glycolysis may result in lactic acidosis, a life-threatening medical condition during sepsis. Therefore, differential induction of dimeric PKM2 expression may be a safer approach to elicit cardioprotection.

A variety of regulatory mechanisms have been proposed to explain PHB2-mediated MQC control, including effects on total protein expression, post-transcriptional phosphorylation, and intracellular compartmentation. A reduction in total PHB2 expression in cardiac-specific PHB2 knockout mice impairs myocardial fatty acid oxidation (FAO) and induces heart failure through suppressing carnitine palmitoyltransferase 1b (CPT1b), a rate-limiting enzyme of cardiac mitochondrial FAO [48]. On the contrary, upregulation of PHB2 expression inhibits cardiomyocyte senescence and therefore delays heart aging through increased mitophagic flux [49]. In addition to altered PHB2 protein expression, phosphorylation of PHB2 at Ser91 is required for myocytic differentiation through MEF2-dependent transcription [44]. Meanwhile, experiments in leukemia cells showed that PHB2 phosphorylation at either S176 or S91 exerts anti-apoptotic actions by attenuating mitochondrial dysfunction [43]. In the nucleus, PHB2 regulates the expression of transcription factors involving in cell cycle progression [42], and increased nuclear PHB2 expression has been regarded as an early event for mitochondrial cristae disarrangement and disruption of inner mitochondrial membrane integrity [50]. Consistent with these findings, we found that experimental SC induces PHB2 downregulation and dephosphorylation in mouse cardiomyocytes. Importantly, our data further suggest that PHB2 dephosphorylation constitutes an endogenous mechanism inducing PHB2 degradation and downregulation. In fact, it is recognized that phosphorylation impacts protein degradation in a site-specific manner, influenced by surrounding amino acid composition, local structure, and phosphoprotein function [51]. In this regard, several proteins, including WRKY [52], p300 [53], c-Myb [54], and RhoA [55], may be degraded through post-transcriptional phosphorylation. Hence, our findings may serve to establish a novel cause-effect relationship between PHB2 phosphorylation and its total expression.

### Limitation

Our present study has a few limitations that deserve mention. Firstly, although we observed that LPS had complex effect on PHB2 expression, additional experiments are required to figure out the mechanism by which LPS induces PHB2 degradation. Secondly, the evaluation of mitochondrial function primarily relied on in vivo experiments, with only a limited number of in vitro experiments analyzing mitochondrial respiration. Consequently, a more comprehensive assessment of mitochondrial function using additional in vitro techniques would strengthen our findings. Besides, the interactive effects between PKM2 and PHB2 were primarily examined in vitro. To better elucidate the underlying interplay mechanism of PKM2-mediated PHB2 phosphorylation, it is essential to conduct animal studies using genetically modified mice. Our present study has a few limitations that deserve mention. Firstly, although we observed that LPS had complex effect on PHB2 expression, additional experiments are required to figure out the mechanism by which LPS induces PHB2 degradation. Secondly, the evaluation of mitochondrial function primarily relied on in vivo experiments, with only a limited number of in vitro experiments analyzing mitochondrial respiration. Consequently, a more comprehensive assessment of mitochondrial function using additional in vitro techniques would strengthen our findings. Besides, the interactive effects between PKM2 and PHB2 were primarily examined in vitro. To better elucidate the underlying interplay mechanism of PKM2-mediated PHB2 phosphorylation, it is essential to conduct animal studies using genetically modified mice. Lastly, it appears that an additional regulatory mechanism is involved in the phosphorylation of PHB2 in *Phb2*^*D/D*^ knock-in mice during LPS exposure, as compared to LPS-treated WT mice and a phosphor-mimetic in vivo mutant seems to be attenuated by LPS due to undefined mechanism. We acknowledge these limitations and believe that addressing them in future studies will provide a more comprehensive understanding of the mechanisms involved.

## Conclusion

In summary, our study highlights a novel mechanism by which PKM2 downregulation impairs MQC in cardiomyocytes during SC. Specifically, our results show that PKM2-mediated phosphorylation of PHB2 at Ser91 is a key upstream event that serves to normalize mitochondrial homeostasis under septic challenge. Our findings may lay the foundation for research and development of new drugs aimed at preventing MQC dysfunction in SC by targeting the PKM2/PHB2 axis.

## Materials and methods

### Septic cardiopathy mouse model

C57BL/6J wild-type (WT) male mice (8–12 weeks old) were obtained from the Jackson Laboratory (Bar Harbor, ME, USA). PKM2 transgenic (PKM2^*Tg*^) mice were generated by Cyagen Biosciences (Santa Clara, CA, USA) according to a previous study [31] with a minor modification. In brief, mouse full-length PKM2 cDNA was amplified, ligated with a pGEMT-Easy vector, and transformed in NEB 5-α competent *E. coli.* Purified transgene constructs were microinjected into C57BL/6J egg pronuclei to generate PKM2^*Tg*^ mice. PHB2^*S91D*^-knockin mice on a C57BL/6 background were also generated by Cyagen Biosciences. All mice were born and maintained under pathogen-free conditions. Male mice were used to induce SC. LPS (*E. coli* 0111: B4, #2630, Sigma-Aldrich, USA) was dissolved in phosphate buffered saline at 10 mg/kg and administered to mice intraperitoneally. Systolic blood pressure was determined by a noninvasive tail-cuff instrument (XBP 1000; Kent Scientific, Torrington, CT, USA). After 24 h, mice were sacrificed and serum was collected to analyze white blood cells (WBC), neutrophils, and monocyte numbers using an automatic blood analyzer (Sysmex XT-2000i; Sysmex Corp., Shanghai, China).

### Echocardiography

Echocardiograms were obtained with a Vevo 2100 Ultrasound System (FUJIFILM VisualSonics Inc., Toronto, Ontario, Canada) equipped with a high-frequency (30 MHz) linear array transducer. Mice were lightly anesthetized with isoflurane (3% for induction and 1.0-1.5% for maintenance) mixed with 1 L/min O_2_ via a facemask [56]. Hair was removed from the chest using a chemical hair remover, and the animals were placed in the left lateral decubitus position. During measurements, body temperature was carefully monitored with a rectal temperature probe and maintained close to 37 °C with the use of a heating pad. Ultrasound gel was applied to the chest and two-dimensional, M-mode, and spectral Doppler images were acquired from modified parasternal long-axis and short-axis views. All images were digitally stored for off-line analysis using Vevo LAB software (version 1.7.1, VisualSonics). Systolic and diastolic anatomic measurements were obtained from M-mode images at the mid-papillary level. LV mass was estimated by the area-length method. Analysis was performed by an investigator who was blind to treatment allocation and unaware of data from other modalities [57].

### Histological analysis and immunohistochemistry

Cardiac mouse tissue was fixed, embedded in paraffin, cut into 4 μm-thick sections, and stained with hematoxylin and eosin (HE). For ICAM1 and VCAM1 immunohistochemistry, 5 μm-thick frozen heart sections were prepared on a cryostat, fixed with methanol/acetone, treated with 3% H_2_O_2_ to quench endogenous peroxidase activity, blocked with 2% bovine serum albumin (BSA), and incubated with anti-VCAM1 (1:500; #ab215380; Abcam, Cambridge, UK) and anti-ICAM1 (1:500; #ab171123; Abcam) antibodies overnight at 4 °C. After extensive washing, sections were incubated with suitable biotinylated secondary antibodies (ABC Kit, MX-200-4; Agilent Technologies, Santa Clara, CA, USA) [58]. Positive staining was visualized using DAB substrate (Solarbio, Beijing, China).

### Immunofluorescence

Heart sections were prepared according to a standard procedure and then incubated with blocking buffer containing 10% (w/v) BSA. Afterward, the sections were incubated overnight at 4 °C with primary antibodies and subsequently with a fluorophore-conjugated secondary antibody (Alexa Fluor® 568 goat anti-rabbit IgG (H + L); A11036; Invitrogen, Waltham, MA, USA) for 1 h. Nuclei were then stained with DAPI and images captured with a fluorescence microscope (BX51, Olympus, Japan). The primary antibodies were listed in the Supplementary Table 1.

### Electron microscopy

Transmission electron microscopy was performed according to a previous report [59]. In brief, samples were fixed in 2.5% glutaraldehyde in 0.1 M sodium cacodylate buffer pH 7.4 for 1 h, and then washed in 100 mM phosphate buffer without CaCl_2_. All samples were post-fixed in 1% osmium tetroxide for 1 h and stained in 2% uranyl acetate in maleate buffer pH 5.2 for an additional hour. The samples were rinsed, dehydrated in an ethanol series, embedded in resin (EMbed 812, EMS, Hatfield, PA, USA), and baked overnight at 60 °C. Hardened blocks were cut using a Leica Ultra Cut UCT microtome. Ultrathin Sect. (60 nm) were collected on carbon-coated grids and contrast-stained using 2% uranyl acetate and lead citrate. Embedded samples were analyzed by a JEM-1400 Plus electron microscope at 80 KV (Jeol Ltd., Tokyo, Japan).

### Primary cardiomyocyte culture and treatment

Mouse hearts were removed and perfused using a Langendorff system for ∼ 3 min with Ca^2+^-free bicarbonate-based buffer. Enzymatic digestion was initiated by adding collagenase type B/D to the perfusion solution. After approximately 7 min, the left ventricle was removed and discarded. Male mice cardiomyocytes from the left ventricular wall were isolated as reported previously by us [60] and plated at 1 ∼ 2 × 10^4^ cells/cm^2^ in mouse laminin pre-coated culture dishes. After 1 h culturing in a 5% CO_2_ incubator at 37 °C, cardiomyocytes were incubated with Fura 2-AM (Invitrogen) for 10 min and then subjected to field stimulation at 2 Hz with platinum electrodes. Contraction and relaxation measures were determined as previously described [59]. Additional experiments were performed using the cardiac mouse HL-1 cell line (American Type Culture Collection, Manassas, VA, USA). To induce inflammation-mediated damage, HL-1 cells were treated with 10 µg/ml LPS for 24 h [35].

### RNA extraction, reverse transcription, and qRT-PCR

Total RNA extraction was performed on heart tissues and HL-1 cells using TRIzol® reagent (Ambion® by Life Technologies, Austin, TX, USA) [14]. Purified RNA samples were reverse transcribed for cDNA synthesis using the GeneAmp™ RNA PCR Core Kit (Applied Biosystems, Waltham, MA, USA). qRT-PCR amplification of target genes and GAPDH (internal control) from heart tissues was performed using the TaqMan® Gene Expression Assay (Applied Biosystems). Amplification of target genes and GAPDH from HL-1 cells was performed using SYBR™ Green qPCR Master Mix (Applied Biosystems). The primers used are listed in Supplementary Table 2. An StepOnePlus™ Fast Real-Time PCR System (Applied Biosystems) was used for all qRT-PCR assays. The *Cytochrome c oxidase subunit I* (CO1) gene of the mtDNA was amplified by qPCR to determine the mtDNA copy. The transcript level of mtDNA was reflected by *NADH dehydrogenase subunit 1* (*ND1*). The *CO1* gene, representing the *cytochrome c oxidase subunit I* in mtDNA, and the *NDUFV1* gene in nDNA were amplified using qPCR. The amplification process began with an initial step at 94 °C for 10 min, followed by 40 cycles consisting of 94 °C for 10 s, 60 °C for 30 s, and 94 °C for 10 s. All reactions were performed in duplicate. Subsequently, the amplification curves were analyzed using SDS 1.9.1 software (Applied Biosystems). Based on a previous study [61], these curves were utilized to determine the relative mtDNA: nDNA ratio in each sample.

### Protein extraction and western blotting

Whole-cell protein lysates were extracted from heart tissues or HL-1 cells using RIPA lysis buffer (150 mM sodium chloride, 1% Triton X-100, 0.5% sodium deoxycholate, 0.1% sodium dodecyl sulfate, 50 mM Tris, pH 8.0) supplemented with Complete Protease and PhosSTOP phosphatase inhibitors (both from Roche Diagnostics, Basel, Switzerland) [62]. To prepare mitochondrial protein lysates, mitochondrial fractions from human HL-1 cells were isolated using the Mitochondria Isolation Kit for Mammalian Cells (Thermo Scientific, Waltham, MA, USA) according to the manufacturer’s protocol. Equal volumes of protein lysates were separated by SDS-PAGE and transferred onto PVDF membranes (GE Healthcare, Little Chalfont, UK). The antibodies used for western blotting are listed in Supplementary Table 1.

### Seahorse assay

The Seahorse XFe96 extracellular flux analyzer (Agilent) was used to assess respiration and mitochondrial function. Cells were seeded on Matrigel-coated assay plates 7 days before measurement at a density of 20,000 cells per XFe96 well. Cells were washed twice in Agilent Seahorse XFe DMEM Basal Medium (Agilent Technologies, cat. no. 103575-100) supplemented with 2 mM glutamine, 10 mM glucose and 1 mM sodium pyruvate 1 h before the assay and for the duration of the measurement. For the standard profiling (Mito Stress test), oligomycin was injected at 1.5 µM, FCCP at 0.5 µM and rotenone/antimycin A were added at 0.5 µM. The oxygen consumption rate (OCR) was normalized to the number of nuclei quantified by DAPI staining. OCR and ATP production rates were obtained using the Seahorse Wave controller Software 2.6.1 (Agilent) as our previously described [63].

### Co-immunoprecipitation

Co-immunoprecipitation (co-IP) was performed as described previously [20]. Briefly, HL-1 cells were harvested and then solubilized with IP buffer (150 mM NaCl, 10% glycerol, 20 mM Tris–HCl pH 7.4, 2 mM EDTA, 0.5% Nonidet P-40, 0.5% Triton X-100, and complete protease inhibitor) for 1.5 h at 4 °C. The insoluble material was removed by centrifugation at 12,000 × g for 10 min, and the supernatant was collected. One-tenth of the supernatant was pipetted out and used as ‘Input’. The remaining supernatant fraction was subsequently incubated with anti-Flag M2 affinity gel (Sigma-Aldrich) at 4 °C overnight. The beads were washed six times with lysis buffer, and the proteins (IP products) were eluted by boiling the beads in SDS sample buffer. Finally, the samples were analyzed by western blotting [64].

### In vitro kinase assay

In vitro kinase assays were conducted at 27 °C for 90 min in 20 mM HEPES pH 7.0, 1 mM TCEP, 10 mM MgCl_2_, 0.1 mM Na_3_VO_4_, and 0.6 µM BSA, with 1.6 µM recombinant mouse PHB2 protein (#MBS1037998; MyBioSource, Inc., San Diego, CA, USA), 12.5 µM ATP, 2 nM recombinant mouse PKM2 protein (#ab95474, Abcam), and 20 nM ADP-heptose or 20 nM DF-006 A, according to our previous reports [65].

### Analysis of myocardial inflammation and myocardial injury markers

Quantitative measurements of myocardial inflammation and myocardial injury biomarkers in cell culture medium or mouse serum were performed using mouse ELISA kits: Serum Creatine Kinase MB (CK-MB) (#E4607; BioVision, Waltham, MA, USA), Troponin I (TNI) (#MBS854284; MyBioSource, Inc.), and Lactate Dehydrogenase (LDH) (#MBS720560; MyBioSource, Inc.) [66]. Apoptosis induction was evaluated with a Caspase-3 Colorimetric Assay Kit (#K106; BioVision, Inc.) and a Caspase-9 Colorimetric Assay Kit (#K119; BioVision, Inc.), as per manufacturer’s instructions [67].

### Mitochondrial membrane potential, mitochondrial ROS, and mitochondrial permeability transition pore (mPTP) detection

For mitochondrial membrane potential quantification, HL-1 cells were incubated in media containing 10 nM JC-1 (#E-CK-A301; Elabscience, Houston, TX, USA) at 37 °C for 10 min. Images were captured under a fluorescence microscope (BX51, Olympus, Japan). MitoSOX™ Red Mitochondrial Superoxide Indicator (#M36008; Thermo Fisher Scientific) was used to evaluate the production of mitochondrial ROS (mROS) in cells under LPS treatment. In brief, cells were incubated with 5 nM MitoSOX™ Red at 37 °C for 10 min and then observed using a fluorescence microscope (BX51, Olympus, Japan). mPTP detection was performed using an Image-IT™ LIVE Mitochondrial Transition Pore Assay Kit (#I35103, Thermo Fisher Scientific) as per manufacturer’s instructions [68].

### Adenovirus vector construction

A PKM2 overexpression plasmid was cloned into pcDNA3.1 (Invitrogen) vector for adenovirus production. PHB2 sequences were translated from HL-1 cell cDNA. PHB2 has four domains, including an N-terminal mitochondrial targeting domain (N, 1–50 aa), a PHB domain (PHB, 68–185 aa), a coiled coil domain (CC, 190–264 aa), and a C-terminal region (C, 265–299 aa). Full length PHB2, PHB2 without N-terminal domain (PHB2ΔN), PHB2 without C-terminal domain (PHB2ΔC), PHB2 without CC domain (PHB2ΔCC), and PHB2 without PHB domain (PHB2ΔPHB) were generated by PCR and ligated into pHAGE-HA vectors for adenovirus production. PKM2 contains an N-terminal domain (N, 1–43 aa), an A domain (44–116 aa and 219–389 aa), a B domain (117–218 aa), and a C region (390–531 aa). Full length PKM2, PKM2 without N domain (PKM2ΔN), PKM2 without A domain (PKM2ΔA), PKM2 without C domain (PKM2ΔC), and PKM2 without N domain (PKM2ΔN) were generated by PCR and ligated into pHAGE-Myc vectors for adenovirus production. The constitutively active (PHB2^S91D^) and inactive (PHB2^S91A^) PHB2 mutant plasmids were obtained to construct lenti-PHB2 viruses as previously described by us [69]. The adenovirus was packaged in 293T cells using Opti-MEM reduced serum medium, along with helper plasmids and PEI reagent. adenovirus supernatant was collected 48 h later and used to infect HL-1 cells after addition of polybrene. Positive clones were then selected by puromycin [69].

### Pulse-chase analysis

Protein stability was determined through pulse-chase analysis as described by us previously [59]. In brief, HL-1 cells were first radiolabeled with [35 S]-Met (100 mCi) under complete medium for the indicated times at 37 °C in the absence or presence of LPS. After washing twice with PBS, cells were cultured under complete medium for the indicated duration. Then, cell samples were lysed and immunoprecipitated with protein G Plus beads coated with the indicated antibodies, followed by western blot analysis [70].

### Cell viability

Cell viability was determined using a Cell Counting Kit-8 (CCK-8) (Dojindo, Kumamoto, Japan) according to the manufacturer’s instructions [71]. HCC cells were seeded in 96-well plates (2 × 10^3^ cells per well) in a final volume of 100 µl. At the indicated time point, 10 µl of CCK-8 reagent was added into each well for 1 h at 37 °C, and absorbance was measured at 450 nm using a multimode plate reader (BioTek, USA).

### Statistical analysis

Data were analyzed using GraphPad Prism 5 software and are shown as mean values and SEM. Differences between two groups were analyzed by unpaired t test if data were normally distributed. Otherwise, Mann-Whitney U test was used. One or two-way ANOVA was used for comparisons between more than two groups. Bonferroni post-hoc test was further performed to compare two specific groups. Two-tailed tests were performed in all statistical analyses, and *p* < 0.05 was considered significant.

### Electronic supplementary material

Below is the link to the electronic supplementary material.

Supplementary Material 1

## Data Availability

The data supporting the findings of this study are found within the article and the supplementary material. All relevant raw data will be made available from the corresponding author upon reasonable request.
