# Supplementary material for: Pyruvate kinase M2 sustains cardiac mitochondrial quality surveillance in septic cardiomyopathy by regulating prohibitin 2 abundance via S91 phosphorylation
Source: Cell Mol Life Sci. 2024 Jun 10;81(1):254. doi: 10.1007/s00018-024-05253-9 (PMC11335292; doi:10.1007/s00018-024-05253-9)
Supplement: Supplementary file 1 — Supplementary Material 1 [file 18_2024_5253_MOESM1_ESM.docx]

Supplemental tables for

**Supplemental Table 1: Antibody information in Western blot**

| Name | Catalogue number | Dilution factor |
| --- | --- | --- |
| Drp1 | Abcam, #ab184247 | 1:1000 |
| Fis1 | Abcam, #ab156865 | 1:1000 |
| Mfn2 | Abcam, #ab124773 | 1:1000 |
| Opa1 | Abcam, #ab42364 | 1:1000 |
| α-tubulin | Cell Signaling Technology, #2144 | 1:1000 |
| Tom20 | Abcam, #ab186735 | 1:1000 |
| PHB2 | Cell Signaling Technology, #14085 | 1:1000 |
| TnT | Abcam, #ab8295 | 1:1000 |
| Gr1 | Abcam, #ab25377 | 1:1000 |
| PKM2 | Abcam, #ab137791 | 1:1000 |
| Bax | Abcam, #ab3191 | 1:1000 |
| LC3II | Abcam, #ab192890 | 1:1000 |
| PGC1α | Abcam, #ab191838 | 1:1000 |
| PHB2 | Abcam, #ab75766 | 1:1000 |
| Myosin | Abcam, #ab254472 | 1:1000 |
| Parkin | Abcam, #ab77924 | 1:1000 |
| Bcl-2 | Abcam, #ab182858 | 1:1000 |
| Beclin1 | Abcam, #ab207612 | 1:1000 |
|  |  |  |

**Supplemental Table 2: Primers for qPCR**

| Gene | Forward Prime | Reverse Prime |  |
| --- | --- | --- | --- |
| *Pgc1α* | 5′-CGGAAATCATATCCAACCAG-3′ | 5′-TGAGGACCGCTAGCAAGTTTG-3′ | |
| *Nrf2* | 5′-CCTCGCTGGAAAAAGAAGTG-3′ | 5′-GGAGAGGATGCTGCTGAAAG-3′ | |
| *Tfam* | 5′-GGCGAATTCCTCGAGGCCACCATG  GCGCTGTTCCGGGGAATGT-3′ | 5′- CATACGCGTATGCTCAGAGATGTC  TCCGGATCGT -3′ | |
| *Gapdh* | 5′-ACGGCAAATTCAACGGCACAGTCA-3′ | 5′-TGGGGGCATCGGCAGAAGG-3′ | |
| *Mmp9* | 5′-CCATCGATTAGAAGCAGGAGGACCCGA-3′ | 5′-GGACTAGTTGGCTAACGCTGCCTTTG-3′ | |
| *Phb2* | 5′-AGCAGGAACAGCACAGAAGA-3′ | 5′-CGGAGCTTGATATAGCCAGGAT-3′ | |
| *Atf5* | 5′-TCCGCTCACACCGTCTCT-3′ | 5′-AAGGCGAAGGTGGAGGAC-3′ | |
| *LonP1* | 5′-GGTTGAGAATGTAGCCCATGA-3′ | 5′-CGATGATATCCCGAATGGTC-3′ | |
| *Pkm2* | 5′-GTCTGGAGAAACAGCCAAGG-3′ | 5′-CGGAGTTCCTCGAATAGCTG-3′ | |
| *Mcp1* | 5'-GGATGGATTGCACAGCCATT-3 | 5'-GCGCCGACTCAGAGGTGT-3' | |
| *Tnfα* | 5'-AGATGGAGCAACCTAAGGTC-3' | 5'-GCAGACCTCGCTGTTCTAGC-3' | |
| *ClpP* | 5′-CACAGACATCGCCATCCA-3′ | 5′-TCCCTCTCCATTGCTGACTC-3′ | |
| *Co1* | 5′-TGCTAGCCGCAGGCATTAC-3′ | 5′-GGGTGCCCAAAGAATCAGAAC-3′ | |
| *ND1* | 5′-CGGCTCCTTCTCCCTACAA-3′ | 5′-ATGGTCCTGCGGCGTATT-3′ | |
| *NDUFV1* | 5'- CTTCCCCACTGGCCTCAAG-3' | 5'-CCAAAACCCAGTGATCCAGC-3’ | |
